# A Core Head, Neck, and Neuroanatomy Syllabus for Physical Therapy Student Education

**DOI:** 10.1002/ca.70016

**Published:** 2025-08-11

**Authors:** Stephanie J. Woodley, Brooke Willoughby, Alexandra L. Webb, Natasha A. M. S. Flack, Laura Y. Whitburn

**Affiliations:** ^1^ Department of Anatomy School of Biomedical Sciences, University of Otago Dunedin New Zealand; ^2^ School of Medicine and Psychology, College of Science and Medicine Australian National University Canberra Australia; ^3^ Department of Microbiology, Anatomy, Physiology and Pharmacology La Trobe University Melbourne Australia

**Keywords:** anatomy, brain, cranial nerves, Delphi, education, head and neck, neuroanatomy, physical therapy, syllabus

## Abstract

Head, neck, and neuroanatomy are essential components of physical therapy education due to their broad clinical applications. Detailed syllabi exist for medical students, yet none have been developed for physical therapy. This study aimed to produce an International Federation of Associations of Anatomists core head, neck, and neuroanatomy syllabus specifically for physical therapy students. A Delphi panel of 45 anatomists and clinicians from 18 countries reviewed 978 head, neck, and neuroanatomy items across five sections: general nervous system; bones and muscles of the head and neck; nasal and oral cavities, pharynx and larynx; the brain; cranial nerves, special senses, and neural pathways (including the autonomic nervous system). Items were rated based on the knowledge required of a minimally competent physical therapy student and categorized as core, recommended, not recommended, or not core. Of the 1001 items in the final topic list, 675 (67%) were rated as core or recommended. For the brain, 85% (311/366) of items were core/recommended, followed by the general nervous system (38/50, 75%) and cranial nerves, special senses, and neural pathways (206/272, 76%). Less than half of the items in the other two categories were considered core/recommended—bones and muscles of the head and neck (108/222, 49%) and nasal and oral cavities, pharynx and larynx (12/91, 13%). This syllabus guides anatomy and physical therapy educators and students in the study of head, neck, and neuroanatomy, emphasizing central nervous system over musculoskeletal and visceral structures.

## 1 Introduction

An in‐depth knowledge of anatomy, that can be applied appropriately to clinical practice, is essential for all health professional students, including those studying physical therapy. A challenge of recent times has been the trend toward decreasing the allocated time, resources, and budgetary support for anatomical education across health professional courses (Bergman et al. 2014; Pan et al. 2020; Rockarts et al. 2020; Sahrmann 2017; Veazey and Robertson 2023), while the amount of required anatomy knowledge has remained the same or increased (Bergman et al. 2014; Drake et al. 2014; Pawlina and Drake 2017). In addition, variability exists in the training of anatomy educators, who come from diverse backgrounds and hold qualifications that may or may not include clinical experience (such as physical therapy) or basic science (i.e., anatomy) knowledge (Bergman et al. 2014; Carroll et al. 2021; Schaefer et al. 2019; Veazey and Robertson 2023). These factors serve to highlight the importance of core anatomy syllabi, which provide some consistency with respect to the breadth and depth of anatomical knowledge expected of health professional students (Moxham et al. 2014; Smith et al. 2020), while also offering flexibility to allow for geographically specific education and alterations when required (Moxham et al. 2014).

Initiated largely by national anatomical societies and the International Federation of Association of Anatomists (IFAA), a number of core anatomy‐focused syllabi have been published to serve as guides to delivering anatomy education. These syllabi encompass a variety of regions and systems such as embryology (Fakoya et al. 2017; Holland et al. 2019), the head and neck (Tubbs et al. 2014), musculoskeletal anatomy (Webb et al. 2019; Woodley et al. 2023), neuroanatomy (Gelb et al. 2021; Moxham et al. 2015), the thorax (Moxham et al. 2020), and gross anatomy in its entirety (Connolly et al. 2018; Finn et al. 2018; Smith et al. 2016). Many of these syllabi have been designed specifically for medical students, with less guidance available for other specialties such as physical therapy, occupational therapy, dentistry, nursing, and pharmacy. With respect to physical therapy, little information exists in relation to anatomy curriculum content (Shead et al. 2020), although some publications have emerged recently that have begun to address this gap. A paper by Pascoe and Rapport (2022) explored 46 anatomy learning objectives across eight body systems to determine which were considered essential in a single entry‐level physical therapy program in the United States of America. Similarly, in a UK‐based study, Gangata et al. (2023) presented 182 learning outcomes that span eight areas, also designed for entry‐level physical therapists. Specific to musculoskeletal anatomy, an IFAA‐commissioned syllabus provides a detailed topic list (with 1700 core or recommended items) that could be used for teaching the vertebral column and limbs to undergraduate physical therapy students (Woodley et al. 2023). Across these publications, some learning outcomes have been described for neuroanatomy (22 in total) (Gangata et al. 2023; Pascoe and Rapport 2022) and the head and neck region (2 in total) (Gangata et al. 2023), but no study has explored specific topic items that may be included in a head, neck, and neuroanatomy physical therapy syllabus.

Neurological conditions are the leading cause of ill health and disability worldwide (Cieza et al. 2021; World Health Organization 2024), with physical therapy playing a central role in facilitating patients' recovery following nervous system injury (Joshua 2022). To ensure accurate clinical decision making, diagnosis, and management, adequate knowledge of nervous system anatomy and function is, therefore, essential for physical therapy students (Singh et al. 2015). Equally, an understanding of head and neck anatomy is, particularly relevant to various areas of physical therapy practice, for example, assessing and managing temporomandibular joint dysfunction, swallowing disorders, or compromised airways (Weden and Haig 2024; Wen et al. 2022).

The aim of this study was to develop a core head, neck, and neuroanatomy syllabus for physical therapy students, following the methodology outlined by the IFAA. For the purpose of this research, a decision was made to integrate neuroanatomy with the head and neck region. Arguably, head and neck anatomy could be integrated into different parts of the curriculum; however, this region is often taught within the same module or course as neuroanatomy (De Louche et al. 2023; Giffin and Drake 2000). Furthermore, brain anatomy is commonly included in textbooks within the head and neck section (e.g., Dalley and Agur 2023; Drake et al. 2020; Schuenke et al. 2020); hence, the decision to combine these topics. Physical therapy students are defined as those who are studying preregistration to become a physical therapist in an undergraduate or graduate‐entry university‐based program.

## 2 Methods

**FIGURE 1:**
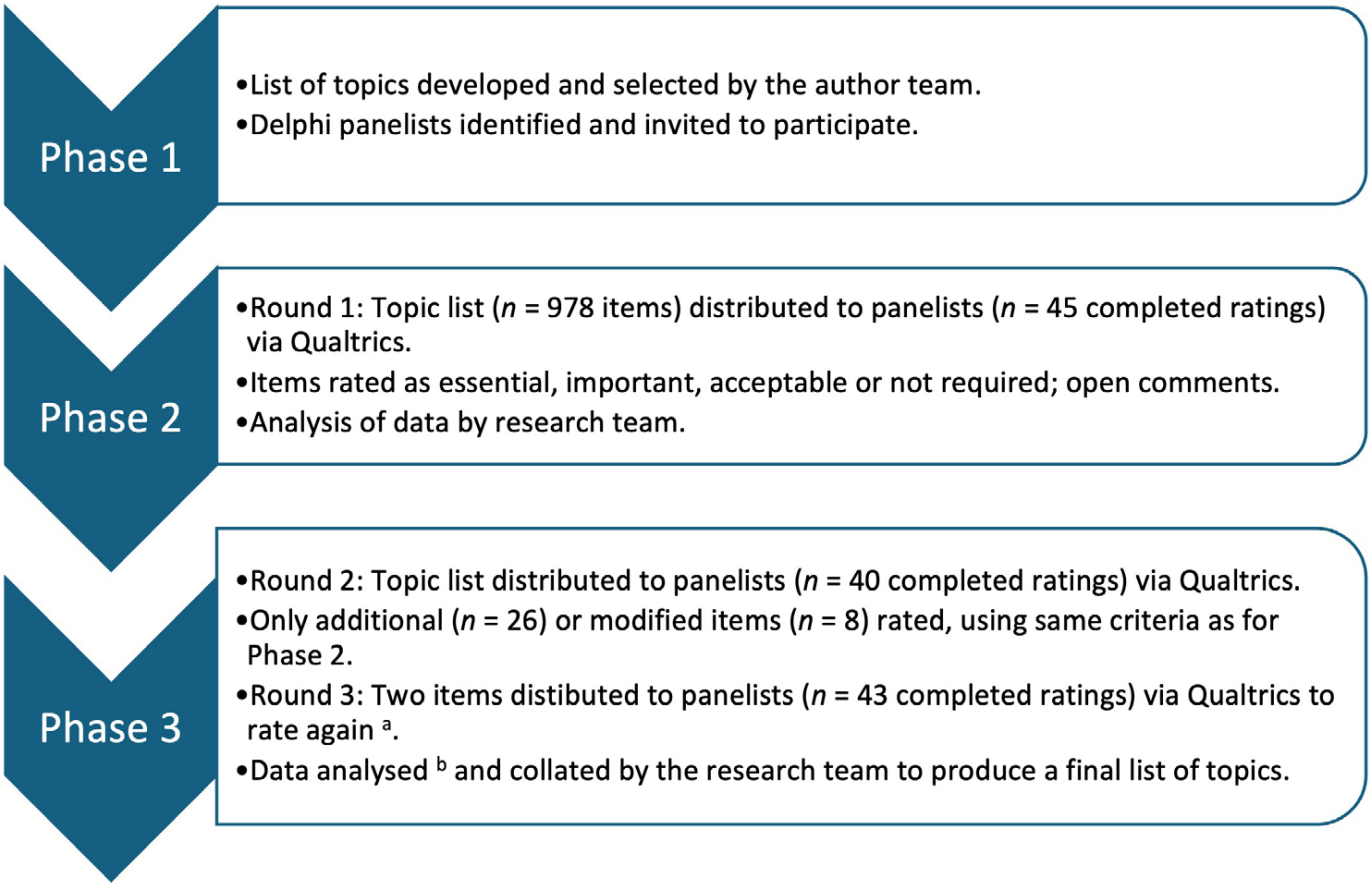
Summary of the first stage of the modified Delphi process. ^a^Items were not classified as essential but were rated important by ≥ 50% of panelists, and so were sent to the panel for further consideration. ^b^Items were categorized as: (1) core: ≥ 60% of panelists considered an item essential; (2) recommended: 30%–59% of panelists classified an item as essential; (3) not recommended: 20%–29% of items rated as essential; and (4) not core: < 20% of items rated essential.

A modified Delphi method was adopted, comprising three stages (Moxham et al. 2014). Stage 1 was the focus of this study and included three phases led by the authors, of whom four (A.L.W., N.A.M.S.F., L.Y.W., S.J.W.) are experienced in physical therapy anatomy education and research (Figure 1) and the fifth was a fourth‐year medical student (B.W.). Following publication of this project, Stages 2 and 3 will involve further dialog between the IFAA and its constituent member organizations (IFAA 2025). Ethics approval for this study was granted by the University of Otago Human Ethics Committee (reference number D22/357).

Phase 1 consisted of compiling the topic items from a range of sources. These sources included the core IFAA syllabi for neuroanatomy and head and neck anatomy, developed for medical students (Moxham et al. 2015; Tubbs et al. 2014), two international anatomical curricula for physical therapy students, and three contemporary gross anatomy textbooks (Drake et al. 2020; Soames and Palastanga 2019; Standring 2021). The list of items was organized into five sections: (1) general nervous system; (2) bones and muscles of the head and neck; (3) nasal and oral cavities, pharynx and larynx; (4) the brain; and (5) cranial nerves, special senses, and neural pathways (including the autonomic nervous system (ANS)).

The Delphi panel was also assembled in Phase 1, with the aim of recruiting anatomists and/or clinicians with expertise in teaching neuroanatomy to physical therapy students, neurological physical therapy, and/or related research. Potential panel members (*n* = 104 from 28 countries) were invited through email either directly or via physical therapy organizations, and by snowball sampling. From this initial invitation, 34 potential panelists did not respond, and 22 were deemed ineligible (e.g., they did not teach neuroanatomy) or were unable to participate. Of the remaining 48 potential panelists, three withdrew before or during Phase 2 due to ill health or because work commitments prevented them from completing the first round of the survey. The final Delphi panel comprised 45 members from 18 countries, with panelists predominantly working as academics and/or clinicians (physical therapists, medical doctors and radiologists) (Table 1). The panelists had been engaged in neuroanatomy teaching and learning for a mean of 14.7 (SD 8.6) years and clinical teaching related to neurology for 10.6 (SD 9.8) years. Many had published papers related to neuroanatomy and/or anatomical education, and some had also authored neuroanatomy textbooks (Table 1).

**TABLE 1:** Demographic characteristics of Delphi panel participants (*n* = 45).

Characteristic	Mean ± SD (min–max)	Percentage
Age (years)	51.9 ± 9.9 (35–69)	
Gender		
Female		62.2
Male		37.8
Primary occupation		
Anatomy academic (teaching and/or research)		44.4
Clinician^a^		2.2
Anatomy academic and clinician^a^		40.0
Other (researcher, senior manager)^b^		13.3
Primary place of work		
University		91.2
Hospital/clinic		4.4
Research institute		0
Other^c^		4.4
Continent/country in which employed		
Africa		11.1
Asia		15.6
Australia and New Zealand		17.8
Europe and the United Kingdom		22.2
America^d^ and Canada		33.3
Time (%) devoted to		
Education	57.7 ± 19.4 (20–100)	
Clinical work	7.6 ± 11.9 (0–60)	
Research	24.3 ± 13.1 (0–50)	
Other (e.g., administration, management)	10.3 ± 11.8 (0–40)	
Authorship (*n*) of		
Neuroanatomy textbook(s)	0.6 ± 1.8 (0–10)	
Neuroanatomy research papers	10.2 ± 21.8 (0–100)	
Pedagogical research papers	17.8 ± 61.8 (0–320)	
Number of years (*n*) involved in		
Delivering teaching and learning of neuroanatomy	14.7 ± 8.6 (1–35)	
Clinical teaching relevant to neuroanatomy	10.6 ± 9.8 (0–35)	
Research relevant to neuroanatomy	9.0 ± 11.1 (0–30)	
Pedagogical research in physical therapy/anatomy education	7.5 ± 8.6 (0–30)	

^a^ Clinicians were physiotherapists (*n* = 12), medical doctors (*n* = 4) or radiologists (*n* = 2).

^b^ Academics specializing in other disciplines such as physical therapy or neuroscience.

^c^ Primary place of work is both university and hospital settings.

^d^ All were from the United States of America, except one panelist who resided in South America.

In Phase 2, an online survey (Qualtrics, Provo, Utah, USA), comprising 978 topic items (organized in five sections) and 13 demographic items, was distributed to the Delphi panelists (Round 1, January 2023) following checking and piloting by the research team and five academics, who were not panel members. Panel members rated each item as essential, important, acceptable, or not required knowledge for a minimally competent physical therapy student (Moxham et al. 2015; Webb et al. 2019; Woodley et al. 2023). Panel members were also asked to include any comments at the end of each group of items, to suggest additional items or amendments, or provide comments on the reasons for their ratings. A timeframe of 4 weeks was provided for panelists to complete the survey, and a reminder email was sent prior to the deadline.

The data were then analyzed by the research team, with items categorized as: (1) core: ≥ 60% of panelists considered the item as essential; (2) recommended: 30%–59% of panelists classified the item as essential; (3) not recommended; and (4) not core: whereby 20%–29% and < 20%, respectively, of items were rated essential (Moxham et al. 2014; Webb et al. 2019). The panel member comments were discussed by the research team and classified as an addition, repetition, modification, justification, or feedback (Webb et al. 2019; Woodley et al. 2023). Suggestions for additional or modified items were returned to the panel for review, and common feedback on specific items, the organization or content of the five sections, or the syllabus in general were noted and included in the discussion section of this manuscript.

In Phase 3, 26 additional items and 7 modified items were rated by the panelists (Round 2, July 2023), and one additional item was modified to rectify a factual error. Following this, two items required further consideration (Round 3, October 2023) as they were not classified as “essential” but were rated “important” by more than 50% of the panel (Webb et al. 2019).

## 3 Results

Forty‐five of the 48 potential panel members participated in Phase 2 (94% response rate) (Table 2). Forty‐four participants completed all sections of the survey (including the demographics section), and one partially completed three of the five sections relating to the different topics; four did not specifically teach head and neck anatomy and therefore only answered four of the sections. In Phase 3 Round 2, 40/45 (89%) of panelists contributed to rating the 33 additional or modified items, and 43/45 (96%) rated the additional two items that required further consideration. In Phase 3 Round 3, 42/45 (93%) rated the two items that required further consideration.

**TABLE 2:** General nervous system.

Topic	Core	Recommended
*Concepts*		
Central nervous system	98%	
Peripheral nervous system	98%	
Anterior (ventral), rostral, caudal, and posterior (dorsal) terminology	64%	
Myelination	62%	
Decussation	62%	
Neuronal regeneration	60%	
Neuronal aging		40%
Neuronal plasticity	64%	
Consciousness		47%
*Connective tissue*		
Endoneurium		36%
Perineurium		40%
Epineurium		42%
*Nerve fiber types*		
General somatic afferent fibers (i.e., afferent fibers for general sensation from somatic structures)	82%	
General visceral afferent fibers (i.e., afferent fibers for general sensation from visceral structures)		51%
Special somatic afferent fibers (i.e., afferent fibers for special senses of sight, hearing, and balance)	73%	
Special visceral afferent fibers (i.e., afferent fibers for special senses of taste and smell)		36%
General somatic efferent fibers (i.e., efferent fibers that innervate skeletal muscle derived from somites)	85%	
Sympathetic general visceral efferent fibers (i.e., efferent fibers for sympathetic stimulation of relevant visceral structures)		38%
Preganglionic sympathetic general visceral efferent fibers		33%
Postganglionic sympathetic general visceral efferent fibers		34%
Parasympathetic general visceral efferent fibers (i.e., efferent fibers for parasympathetic stimulation of relevant visceral structures)		44%
Preganglionic parasympathetic general visceral efferent fibers		31%
Postganglionic parasympathetic general visceral efferent fibers		31%
Special visceral efferent/pharyngeal efferent fibers (i.e., efferent fibers that innervate skeletal muscle derived from pharyngeal arches)		42%
Interneurons		51%
*Pathology*		
Multiple sclerosis	71%	
Guillain–Barré syndrome		51%
Neuropraxia		53%
Axonotmesis		53%
Neurotmesis		51%
Spina bifida		47%
Meningocele		36%
Cerebral palsy	71%	
Myasthenia gravis		47%
Spinal cord injury/syndrome	75%	
Vascular injury/condition		50%
Differentiate between an upper motor neuron and lower motor neuron lesion	78%	
Common gait anomalies associated with nervous system		55%

After the ratings had been analyzed, 1001 items were included in the final topic list, with 675 (67%) rated as core or recommended. Across the five topic sections, the number of items classified as core or recommended was: (1) general nervous system (*n* = 38, Table 2); (2) bones and muscles of the head and neck (*n* = 108, Table 3); (3) nasal and oral cavities, pharynx and larynx (*n* = 12, Table 4); (4) the brain (*n* = 311, Table 5); and (5) cranial nerves, special senses, and neural pathways (*n* = 206, Tables 6 and 7). The following information summarizes the findings across all sections and includes a description of the additions and modifications (in Section 3.6). The full topic list with the final ratings is presented in the Supporting Information, with anatomical terminology adhering to the recommendations of the Federative International Programme for Anatomical Terminology (FIPAT 2019).

**TABLE 3:** Bones and muscles of the head and neck.

Topic	Core	Recommended
*Bones of the skull—neurocranium*		
Frontal bone	69%	
Orbital cavity		38%
Parietal bone	71%	
Temporal bone	69%	
Internal acoustic meatus		36%
External acoustic meatus		38%
Zygomatic process of temporal bone		44%
Mastoid process		51%
Styloid process of temporal bone		38%
Articular tubercle		36%
Mandibular fossa		51%
Stylomastoid foramen		34%
Jugular foramen		41%
Occipital bone	69%	
Foramen magnum	60%	
External occipital protuberance		47%
Superior nuchal line		40%
Inferior nuchal line		35%
Occipital condyle		51%
Hypoglossal canal		33%
Sphenoid bone	60%	
Body of sphenoid bone		36%
Greater wing		33%
Lesser wing		31%
Optic foramen		36%
Optic canal		36%
Pituitary fossa		36%
Foramen rotundum		36%
Foramen ovale		36%
Carotid canal		36%
Superior orbital fissure		42%
Inferior orbital fissure		33%
Ethmoid bone		56%
Cribriform plate		38%
Cranial fossae	60%	
Anterior fossa		47%
Middle fossa		49%
Posterior fossa		49%
Cerebellar fossa		42%
*Neurocranium—concepts*		
Intramembranous ossification process		33%
Function of skull bones (e.g., protection of brain)	71%	
Surface anatomy and palpation of the head and neck		53%
*Neurocranium—pathology*		
Fracture at/near pterion		36%
*Joints—cranial sutures*		
Joint type: fibrous suture (dense fibrous connective tissue)	62%	
Movement: Little to no movement	62%	
Coronal suture		40%
Sagittal suture		40%
Lambdoid suture		40%
Bregma		31%
Pterion		40%
Purpose of fontanelles		47%
*Bones of the skull—facial skeleton*		
Maxilla	74%	
Hard palate		31%
Lacrimal		40%
Zygomatic bone	60%	
Zygomatic arch		43%
Mandible	72%	
Angle of mandible		52%
Neck of mandible		45%
Body of mandible		48%
Ramus of mandible		50%
Mandibular foramen		33%
Mandibular notch		31%
Mandibular condyle		50%
Coronoid process of mandible		50%
Vomer		36%
Palatine bone		43%
Nasal bone		45%
Hyoid bone		55%
*Joints—temporomandibular joint*		
Classification: see text		
Movements: retraction/retrusion, protraction/protrusion, depression and elevation and lateral deviation	79%	
Lateral temporomandibular ligament		53%
Sphenomandibular ligament		43%
Stylomandibular ligament		43%
Articular disc of temporomandibular joint	69%	
Articular surfaces lined by fibrocartilage		57%
Functional concept: maximum stability of the temporomandibular joint is when the mouth is closed and teeth occluded		55%
Functional concept: superior and inferior joint cavities of the temporomandibular joint move in different ways to produce different movements (e.g., superior produces gliding, inferior produces hinge‐like movements)		60%
*Facial skeleton—pathology*		
Temporomandibular joint disorders		50%
*Muscles of the head and neck*		
Primary muscles of mastication	77%	
Medial pterygoid muscle	70%	
Lateral pterygoid muscle	70%	
Masseter	77%	
Temporalis muscle	77%	
Muscles of facial expression		38%
Occipitalis muscle		41%
Frontalis muscle		50%
Orbicularis oculi muscle		43%
Orbicularis oris muscle		43%
Bucinator		50%
Muscles of the eye		48%
Extraocular muscles		52%
Superior oblique muscle		45%
Inferior oblique muscle		45%
Superior rectus muscle		43%
Inferior rectus muscle		43%
Medial rectus muscle		43%
Lateral rectus muscle		45%
Suprahyoid muscles		30%
Infrahyoid muscles		30%
*Muscles of the head and neck—functional concepts*		
Muscles of mastication—allow for chewing and grinding motions	73%	
Muscles of facial expression—have a role in opening or closing mouth, eyes, and nose	71%	
Muscles of facial expression—have a role in expression of emotions	67%	
Unilateral and bilateral muscle contractions produce different movements at the temporomandibular joint	61%	
Synergistic activity of the pterygoid muscles produces different movements at the temporomandibular joint		56%
Voice—controlled by contractions of the laryngeal muscles		39%
Swallowing—controlled by muscles of the pharynx and tongue		44%
Mechanical digestion—muscles of the tongue contribute to this		37%
Muscles of the eye allow for change in pupil size		51%

**TABLE 4:** Nasal and oral cavities, pharynx, and larynx.

Topic	Core	Recommended
*Nasal cavity—concepts*		
Function of nasal cavity		37%
Regions for olfaction and respiration		32%
*Pharynx*		
Pharynx		46%
Nasopharynx		39%
Oropharynx		39%
Laryngopharynx		39%
*Oral cavity*		
Mouth		44%
Tongue		46%
Salivary glands		39%
*Larynx*		
Larynx		46%
Thyroid cartilage		32%
Glandular structures		34%

**TABLE 5:** The brain.

Topic	Core	Recommended
*Cerebrum*		
Gray matter	91%	
Cerebral cortex	89%	
Layers of the cerebral cortex		39%
White matter	89%	
Commissural fibers	66%	
Association fibers	62%	
Projection fibers	66%	
Corpus callosum	73%	
Rostrum of corpus callosum		41%
Genu of corpus callosum		39%
Body of corpus callosum		41%
Splenium of corpus callosum		36%
Septum pellucidum		32%
Corona radiata		55%
Major sulci and fissures	84%	
Central sulcus	84%	
Longitudinal cerebral fissure	78%	
Transverse cerebral fissure		57%
Lateral sulcus	77%	
Parieto‐occipital sulcus	64%	
Frontal lobe	91%	
Primary functions: voluntary motor function, language, planning, mood, smell and social judgment, personality, intellect, and complex learning abilities	89%	
Precentral sulcus		59%
Precentral gyrus	75%	
Superior frontal sulcus		34%
Inferior frontal sulcus		32%
Superior frontal gyrus		39%
Middle frontal gyrus		39%
Inferior frontal gyrus		41%
Opercular part of inferior frontal gyrus		32%
Triangular part of inferior frontal gyrus		30%
Orbital part of inferior frontal gyrus		30%
Parietal lobe	84%	
Primary functions: somatosensory perception and integration of sensory information	86%	
Postcentral sulcus	66%	
Postcentral gyrus	82%	
Intraparietal sulcus		34%
Superior parietal lobule		34%
Inferior parietal lobule		34%
Supramarginal gyrus		41%
Angular gyrus		41%
Temporal lobe	93%	
Primary functions: Hearing, smell, learning, memory, fear, and emotion	86%	
Transverse temporal gyri		39%
Superior temporal gyrus		34%
Occipital lobe	86%	
Primary function: vision	86%	
Preoccipital notch		34%
Calcarine sulcus		55%
Cuneus		30%
Lingual		30%
Insular lobe	73%	
Primary functions: play a role in avoidance learning, decision‐making, emotions and possibly addiction	68%	
*Cerebrum—functional areas*		
Primary functional areas	93%	
Primary motor cortex	91%	
Primary somatosensory cortex	91%	
Primary auditory area	87%	
Primary visual cortex	89%	
Secondary functional areas	82%	
Premotor cortex	84%	
Supplementary motor areas	80%	
Broca area	89%	
Function of Broca area: center for expressive (motor) speech	86%	
Secondary somatosensory cortex		57%
Secondary auditory areas		48%
Secondary visual areas		50%
Association areas	73%	
Association somatosensory areas	66%	
Wernicke area	86%	
Function of Wernicke area: permits comprehension of spoken and written language and creates plans for formulation of speech	89%	
Prefrontal cortex	82%	
*Cerebrum—pathology*		
Lesions of the primary visual cortex		57%
Lesions of the secondary visual area		34%
Lesions of the primary auditory area		48%
Lesions of the secondary auditory area		34%
Lesions of the prefrontal cortex	62%	
Lesions of the primary somatosensory cortex	68%	
Lesions of the secondary somatosensory cortex		43%
Lesions of the association somatosensory area		43%
Lesions of the primary motor cortex	82%	
Lesions of the supplementary motor area		57%
Lesions of the premotor cortex		59%
Epilepsy		34%
Aphasia (nonfluent)—lesion of Broca area		52%
Aphasia (fluent)—lesion of Wernicke area		52%
Apraxia		59%
Astereognosis		48%
Neglect syndrome (spatial neglect)	62%	
Contralateral homonymous hemianopia		57%
Dysarthria		50%
Tonic spasm		39%
Dysphagia		45%
Alzheimer's disease		55%
Dementias		50%
Brain tumors		48%
Cerebral edema		52%
Traumatic brain injury		55%
Concussions		59%
Amyotrophic lateral sclerosis		48%
Migraines		36%
Headaches		41%
*Diencephalon*		
Thalamus	89%	
Function of the thalamus	82%	
Nuclei of the thalamus		32%
Medial and lateral geniculate bodies		34%
Hypothalamus	80%	
Function of the hypothalamus	75%	
Mammillary body		32%
Optic chiasm		59%
Pineal gland		50%
Function of the pineal gland		45%
Subthalamus		45%
Subthalamic nuclei		41%
Pituitary gland		57%
Adenohypophysis		30%
Neurohypophysis		30%
Neuroendocrine function		36%
*Diencephalon—concepts*		
Arrangements and connections of the diencephalon		46%
Function—Sensory integration		50%
*Basal nuclei/ganglia*		
Caudate nucleus	70%	
Head of caudate nucleus		32%
Body of caudate nucleus		32%
Tail of caudate nucleus		32%
Lentiform nucleus	68%	
Putamen	61%	
Globus pallidus	61%	
Substantia nigra	75%	
Internal capsule	77%	
Functions of the internal capsule	73%	
Genu of internal capsule		48%
Posterior limb of internal capsule		45%
Anterior limb of internal capsule		43%
*Basal nuclei/ganglia—concepts*		
Functions of the basal nuclei	89%	
Circuits between the basal nuclei, cerebral cortex, and cerebellum	66%	
Input nuclei of the basal nuclei		48%
Intrinsic nuclei of the basal nuclei		45%
Output nuclei of the basal nuclei		48%
*Basal nuclei/ganglia—pathology*		
Parkinson disease	80%	
Huntington disease		57%
Hypokinesia		59%
Hyperkinesia		57%
Chorea	61%	
Athetosis		55%
Hemiballismus		52%
Tic		41%
Resting tremor		59%
*Limbic system*		
Hippocampus	77%	
Function: associated with formation of long‐term memories	77%	
Hippocampal formation		36%
Amygdalaloid body	68%	
Function: signaling the cortex of motivational stimuli	66%	
Amygdaloid nuclei		30%
Parahippocampal gyrus		50%
Function: associated with memory formation		48%
Cingulate gyrus	64%	
Functions: autonomic functions which regulate heart rate, blood pressure, and processes such as cognition and attention		57%
Fornix		34%
Function: carries signals from the hippocampus to the mammillary bodies		30%
Dentate gyrus		36%
Function: new memory formation and regulation of mood		34%
*Limbic system—concepts*		
Connecting pathways of the limbic system		41%
The role of the limbic system in emotion		30%
*Brainstem*		
Midbrain	86%	
Cerebral peduncle		55%
Crus cerebri		52%
Interpeduncular fossa		41%
Superior colliculus		55%
Inferior colliculus		55%
Superior cerebellar peduncle		55%
Red nucleus		43%
Tectum of midbrain		43%
Pons	82%	
Basilar part of pons		39%
Middle cerebellar peduncle		50%
Main sensory nucleus of trigeminal nerve		48%
Medulla oblongata	91%	
Pyramid of medulla oblongata	82%	
Inferior cerebellar peduncle		55%
Anterior median fissure of medulla oblongata		34%
Decussation of pyramids	80%	
Olive		54%
Olivary nuclei		43%
Vestibulocochlear nuclei		48%
Gracile nucleus		52%
Cuneatus nucleus		52%
Gracile fasciculus	61%	
Cuneate fasciculus	61%	
Spinal nucleus of trigeminal nerve		50%
Reticular formation	71%	
Reticular activating system nuclei		33%
Functions of the reticular formation		59%
*Brainstem—concepts*		
Associations of brainstem with descending and ascending tracts to and from spinal cord and cerebral cortex	91%	
Associations of brainstem with cerebellum	91%	
Tracts which originate in brainstem	77%	
*Brainstem—pathology*		
Lesions of the midbrain		48%
Lesions of the pons		43%
Lesions of the medulla oblongata		46%
Lesions of the reticular formation		43%
Loss of consciousness		52%
Myoclonus		34%
*Cerebellum*		
Gray matter	80%	
Cerebellar cortex	75%	
Layers of cerebellar cortex		43%
Hemisphere of cerebellum	77%	
Lateral hemisphere	66%	
Function of lateral hemisphere: integrative, projections to motor/premotor cortex	71%	
Vermis of cerebellum	61%	
Function of vermis: responds to proprioceptive and somatosensory input	61%	
Cerebellar lobes	68%	
Anterior lobe of cerebellum		52%
Function of anterior lobe: Important in movement coordination	66%	
Middle lobe of cerebellum		55%
Function of middle lobe: Important in movement coordination	66%	
Flocculonodular lobe		52%
Function of flocculonodular lobe: Important for adjustments of posture to maintain balance and vestibular functions	64%	
Primary fissure		34%
Flocculus		39%
Nodule of vermis		39%
Tonsil of cerebellum		43%
White matter	64%	
Arbor vitae		34%
*Cerebellum—pathology*		
Ataxia	75%	
Dysmetria	66%	
Dysdiadochokinesia	66%	
Dyssynergia		57%
Reflex disturbances		57%
Postural and gait changes	73%	
Ocular movement disturbances		59%
Disorders of speech		45%
Intention tremor	64%	
*Meninges*		
Dura	86%	
Periosteal cranial dura		45%
Meningeal cranial dura		46%
Layers are fused except for where they split to form venous sinuses		48%
Falx cerebri		55%
Falx cerebelli		50%
Tentorium cerebelli		55%
Subdural space	64%	
Arachnoid	82%	
Arachnoid granulations		55%
Subarachnoid space	68%	
Pia	80%	
*Meninges—pathology*		
Subdural hematoma	66%	
Extradural hemorrhage	64%	
Subdural hemorrhage	61%	
Subarachnoid hemorrhage	61%	
Intracranial hemorrhage	66%	
Movements of brain in relation to meninges in head injury		57%
Meningitis		34%
*Ventricular system*		
Cerebrospinal fluid	84%	
Formation of cerebrospinal fluid		55%
Circulation of cerebrospinal fluid		57%
Absorption of cerebrospinal fluid		52%
Choroid plexus		50%
Ventricles	77%	
Lateral ventricle	70%	
Frontal horn		32%
Body of lateral ventricle		32%
Occipital horn		30%
Temporal horn		30%
Third ventricle	70%	
Interventricular foramen	61%	
Aqueduct of midbrain (cerebral aqueduct)	66%	
Fourth ventricle	70%	
Median aperture of fourth ventricle		32%
Lateral aperture of fourth ventricle		32%
Posterior cerebellomedullary cistern (cisterna magna)		34%
*Ventricular system—pathology*		
Changes in intracranial pressure		35%
Hydrocephalus		55%
Raised cerebrospinal fluid pressure		50%
*Blood supply*		
Arteries	89%	
Common carotid artery	77%	
Internal carotid artery	84%	
External carotid artery	68%	
Vertebrobasilar system	82%	
Anastomoses	71%	
Cerebral arterial circle (circle of Willis)	91%	
Anterior cerebral artery	82%	
Middle cerebral artery	86%	
Posterior cerebral artery	84%	
Anterior communicating artery	75%	
Posterior communicating artery	75%	
Basilar artery	77%	
Anterior inferior cerebellar artery	64%	
Superior cerebellar artery	62%	
Posterior inferior cerebellar artery	64%	
Middle meningeal artery		36%
Veins	68%	
Superior sagittal sinus		57%
Inferior sagittal sinus		50%
Occipital sinus		34%
Transverse sinus		48%
Straight sinus		38%
Cavernous sinus		34%
Confluence of sinuses		36%
Sigmoid sinus		36%
Internal jugular vein		54%
Lymphatics		54%
Thoracic duct		41%
*Blood supply—pathology*		
Cerebral ischemia	77%	
Cerebral infarction	77%	
Cerebral aneurysm	66%	
Congenital aneurysm		36%
Postural hypotension		54%
Hypotension		57%
Hypertension	61%	
Diseases that alter blood pressure interrupting cerebral circulation		50%
Ischemic stroke	75%	
Hemorrhagic stroke	75%	
Transient ischemic attack	71%	
Lymphadenopathy		30%

**TABLE 6:** Cranial nerves and special senses.

Topic	Core	Recommended
*Cranial nerves*		
Olfactory nerve (I)	64%	
Function: special sensory—smell	64%	
Optic nerve (II)	73%	
Function: special sensory—vision	73%	
Pathway: ganglion cells of retina to visual processing areas		52%
Central connections of the optic nerve		45%
Oculomotor nerve (III)	70%	
Function: motor to extraocular muscles and parasympathetic motor to control pupil	68%	
Pathway: ventral midbrain at level of superior colliculus to site of action		48%
Trochlear nerve (IV)	68%	
Function: motor to superior oblique muscle	66%	
Pathway: dorsal aspect of midbrain below inferior colliculus to site of action		46%
Trigeminal nerve (V)	82%	
Function: sensation from face and motor to muscles of mastication	82%	
Pathway: trigeminal ganglion to junction of pons and middle cerebellar peduncle (sensory), same junction to site of action (motor)		52%
Ophthalmic nerve—sensation from upper third of face	66%	
Maxillary nerve—sensation from middle third of face	66%	
Mandibular nerve—sensation from lower third of face and somatic motor to muscles of mastication	66%	
Abducens nerve (VI)	70%	
Function: motor to lateral rectus muscle	68%	
Pathway: junction of pons and pyramid of medulla oblongata to site of action		41%
Facial nerve (VII)	86%	
Function: motor to muscles of facial expression, parasympathetic motor to submandibular and sublingual salivary and lacrimal glands; sensation from skin of auricle; and special sensory for taste from anterior 2/3rds of tongue	86%	
Pathway: geniculate ganglion (sensory) to lateral edge of pontomedullary junction, same junction to site of action (motor)		50%
Vestibulocochlear nerve (VIII)	82%	
Function: special sensory for hearing and balance	80%	
Pathway: vestibular/spiral ganglion to lateral edge of the pontomedullary junction		45%
Glossopharyngeal nerve (IX)	73%	
Function: motor to pharyngeal muscles for swallowing, parasympathetic motor to parotid gland; sensory from posterior 1/3rd of tongue, posterior auricle, tragus, soft palate, pharynx, tympanic membrane and cavity, pharyngo‐tympanic tube, mastoid cells, carotid bodies and sinus; special sensory for taste from posterior 1/3rd of tongue)	68%	
Pathway: medulla to sites of action (motor), ganglia for taste and carotid bodies and sinus to medulla (sensory)		34%
Vagus nerve (X)	86%	
Function: motor to muscles of the pharynx, larynx and soft palate; parasympathetic motor to smooth muscle of digestive and respiratory tracts and cardiac muscle; special sensory for taste from epiglottis and palate; and sensation from thoracic and abdominal viscera, carotid sinus and carotid and aortic bodies, auricle, external acoustic meatus, and dura mater of posterior cranial fossa	82%	
Pathway: lateral medulla to sites of action (motor), ganglion of CNX to lateral medulla (sensory)		48%
Accessory nerve (XI)	84%	
Function: motor to larynx (cranial root) and sternocleidomastoid and trapezius (spinal root)	82%	
Pathway: cranial from lateral medulla posterior to olives, spinal from supraspinal nucleus to sites of action		45%
Spinal root of accessory nerve		36%
Hypoglossal nerve (XII)	68%	
Function: motor to intrinsic and extrinsic muscles of the tongue (except palatoglossus—CN X)	66%	
Pathway: rootlets between olive and pyramid of medulla oblongata to sites of action		30%
*Cranial nerves—pathology*		
Trigeminal neuralgia		50%
Lesions of the visual pathway		50%
Lesions of the oculomotor, trochlear and abducens nerve		43%
Facial nerve lesions and Bell palsy	64%	
Vertigo		55%
Nystagmus		48%
*Cranial nerves—examination*		
Smell test—CN I		36%
Confrontation test—CN II		39%
Light reflexes—CN III		45%
Accommodation reflexes—CN III		43%
Corneal reflex—CN V		36%
Jaw jerk reflex—CN V		34%
Facial sensation—CN V		50%
Blink reflex—CN VII		36%
Hearing test—CN VIII		36%
Balance test—CN VIII		53%
Elevation of soft palate—CN IX and X		32%
Cough—CN X		39%
Test sternocleidomastoid—CN XI		52%
Tongue protrusion—CN XII		43%
*Special senses—vision*		
Orbit		43%
Eye/eyeball		45%
Cornea		34%
Retina		43%
Lens		34%
Optic disc		30%
Macula		32%
Fovea centralis		32%
*Special senses—vision—pathology*		
Diabetic retinopathy		30%
*Special senses—hearing and balance*		
Ear		59%
External ear		50%
Auricle		39%
Middle ear		59%
Tympanic membrane	64%	
Malleus		41%
Incus		41%
Stapes		41%
Auditory tube		45%
Vestibular window		36%
Internal ear		59%
Bony labyrinth		48%
Vestibule		55%
Utricle		48%
Saccule		48%
Maculae		41%
Semicircular canals	61%	
Semicircular ducts		52%
Ampulla of semicircular ducts		41%
Perilymph		41%
Cochlear		37%
Cochlear duct		34%
Cochlear ganglion		30%
Cochlear nerve		45%
Vestibular apparatus		52%
Vestibular ganglia		32%
Vestibular nuclei		43%
Vestibulo‐ocular reflex		59%
*Special senses—hearing and balance—pathology*		
Vertigo		50%

**TABLE 7:** Neural pathways.

Topic	Core	Recommended
*Motor control*		
Motor neurons	98%	
Upper motor neuron (cell body located in the brainstem or cortex; does not have an axon in the peripheral nervous system)	93%	
Lower motor neuron (cell body located in the brainstem or spinal cord; has an axon in the peripheral nervous system innervating muscle)	93%	
Pyramidal motor pathways	93%	
Corticospinal tract	93%	
Anterior corticospinal tract	84%	
Lateral corticospinal tract	91%	
Function: motor control of skeletal muscles in the body	93%	
Pathway: cerebral cortex to spinal cord	91%	
Corticonuclear fibers (corticobulbar tract)	82%	
Function: motor control of skeletal muscles of face, head and neck	82%	
Pathway: cerebral cortex to brainstem	80%	
Extrapyramidal motor pathways	75%	
Tectospinal tract	61%	
Function: reflexive postural movements in response to visual stimuli	64%	
Pathway: from superior colliculus of midbrain to spinal cord		55%
Vestibulospinal tracts	70%	
Function: facilitate activity of extensor muscles and inhibits activity of flexor muscles to contribute to the maintenance of balance	68%	
Pathway: from vestibular nuclei in medulla and pons to spinal cord	61%	
Rubrospinal tract		59%
Function: facilitates activity of flexor muscles and inhibits activity of extensor or antigravity muscles	64%	
Pathway: from red nucleus to spinal cord		52%
Reticulospinal tract	64%	
Function: may facilitate or inhibit voluntary movement and reflex activity, as well as control sympathetic and parasympathetic outflow	64%	
Pathway: from pontine reticular formation to spinal cord		55%
Descending fibers from all areas of cortex through the crus cerebri to pons; with decussation into cerebellum via the middle peduncle		35%
*Motor control—concepts*		
Voluntary movement occurs in three steps: planning, programming and execution	93%	
Motor homunculus in primary motor cortex	91%	
Spinal reflex arc	93%	
Neuromuscular junction	91%	
Motor unit	89%	
Open and closed loop control	75%	
Clinical examination of motor control (e.g., testing spinal reflexes, muscle tone and spasticity, coordination etc.)	84%	
*Motor control—pathology*		
Upper motor neuron lesions and their consequences	89%	
Lower motor neuron lesions and their consequences	89%	
Paresis	77%	
Hemiparesis	77%	
Hemiplegia	77%	
Paralysis	77%	
Spasticity	77%	
Rigidity	77%	
Flaccidity	77%	
Hypotonia	77%	
Hypertonia	77%	
Dystonia	75%	
Tremors	73%	
Muscle atrophy	80%	
*Sensation*		
Sensory neurons	98%	
First order (primary) afferents (i.e., sensory neurons with cell body located in dorsal root ganglion)	91%	
Second order (secondary) afferents (i.e., sensory neurons with cell body located in spinal cord/brainstem with axons projecting into the thalamus)	87%	
Third order afferents (i.e., sensory neurons with cell bodies located in thalamus with axons projecting to sensory cortex)	87%	
Medial lemniscus/dorsal column pathway	96%	
Function: carries discriminative sensation (discriminatory touch and vibration), and conscious proprioception	93%	
Pathway: gracile fasciculus carries input from lower half of body to gracile nucleus and cuneate fasciculus carries input from upper half of body to cuneate nucleus	82%	
Lateral spinothalamic tract	93%	
Function: carries nociception and temperature	89%	
Pathway: to thalamus along lateral spinothalamic tracts	77%	
Anterior spinothalamic tract	84%	
Function: carries crude touch and pressure	80%	
Pathway: to thalamus along anterior spinothalamic tract	68%	
Spinocerebellar tracts	77%	
Function: carry unconscious proprioceptive information	75%	
Pathway: along posterior spinocerebellar tract which passes through the posterior thoracic nucleus and accessory cuneate nucleus or anterior spinocerebellar tract to cerebellum		55%
Cuneocerebellar tract		
Function: carries unconscious proprioceptive information from the upper limbs		30%
Posterior spinocerebellar tract		33%
Function: carries unconscious proprioceptive information from the trunk and lower limbs		35%
Sensory receptors	75%	
Mechanoreceptor—Meissner corpuscles		52%
Function of Meissner corpuscles: sense fine touch or discriminative sensation		57%
Mechanoreceptor—Pacinian corpuscles		57%
Function of Pacinian corpuscles: sense pressure and vibration		59%
Free nerve terminals	61%	
Function of free nerve terminals: thermoreception (temperature) and nociception	66%	
Proprioceptive and position sense receptor—muscle spindles	68%	
Intrafusal muscle fibers	64%	
Proprioceptive and position sense receptor—Golgi tendon organs	70%	
Proprioceptive and position sense receptor—joint receptors	70%	
*Sensation—concepts*		
Sensory modalities (e.g., discriminatory and crude touch, conscious and unconscious proprioception, nociception, vibration sense, etc.)	89%	
Conscious (e.g., discriminatory touch) and unconscious (e.g., unconscious proprioceptive) sensory information	84%	
Pain	89%	
Somatic pain	82%	
Visceral pain	75%	
Pain versus nociception	80%	
Peripheral and central sensitization	73%	
Pain as a protective output of the CNS	73%	
Relationship between neural plasticity and persistent pain states	73%	
Sensory homunculus in primary somatosensory cortex	82%	
Clinical examination of sensation	75%	
*Sensation—pathology*		
Understand lesions of sensory pathways and their consequences	89%	
Persistent pain	70%	
Phantom limb	64%	
Referred pain	77%	
*Somatic plexi*		
Cervical plexus— please note, all other plexuses have been included in the musculoskeletal syllabus		57%
*Autonomic nervous system*		
Nerve plexi		39%
Parasympathetic nervous system	70%	
Preganglionic cell bodies associated with CN III, VII, IX, and X and lateral horns of S2–S4 segments of spinal cord		55%
Postganglionic cell bodies within or close to target organ		41%
Sympathetic nervous system (SNS)	75%	
Preganglionic cell bodies in lateral horns of T1‐L2 segments of spinal cord		55%
Sympathetic trunk		50%
Paravertebral ganglia		39%
Prevertebral ganglia		36%
Pathways for SNS to exit the sympathetic trunk		32%
*Autonomic nervous system—concepts*		
General organization of the autonomic nervous system, i.e., sympathetic and parasympathetic divisions	80%	
Autonomic innervation of the body	77%	
Autonomic nervous system functions to maintain homeostasis	77%	
*Autonomic nervous system—pathology*		
Autonomic control following spinal cord injury	62%	
Intermittent claudication		39%

Abbreviation: CN = cranial nerve.

### 3.1 General Nervous System

Thirty‐eight of 50 (75%) items in this section were considered core/recommended (Table 2). All nine general concepts relating to the nervous system (such as system components, terminology, neuronal organization and changes over time, and consciousness), as well as the three types of connective tissue associated with the nervous system, and all 13 items relating to nerve fiber types, were considered core/recommended. In addition, 13/16 items relating to pathology attained a classification of core/recommended. In contrast, none of the nine items relating to the development of the nervous system were considered core/recommended.

### 3.2 Bones and Muscles of the Head and Neck

Of the 222 items in this category, 49% (108) were considered core/recommended (Table 3). The bones of the skull and face were all rated core/recommended (14/14) but only 41% (38/92) of their bony landmarks. All of the articular structures of the skull were regarded as core/recommended, including cranial sutures (8/8) and the temporomandibular joint (8/8). The majority of panel members designated the temporomandibular joint as synovial modified hinge (50%) or synovial condylar (45%) instead of synovial ball and socket (5%).

Half of the muscle groups of the head and neck (6/12) were considered core/recommended, including the muscles of mastication, facial expression, eye, extraocular, and suprahyoid and infrahyoid muscles. Only 28% of individual muscles were specified as core/recommended (15/54) and included the primary muscles of mastication (4/4), facial expression (5/20), and extraocular (6/6) groups. All functional concepts related to the muscles of the head and neck were ranked core/recommended (9/9).

### 3.3 Nasal and Oral Cavities, Pharynx, and Larynx

Only 12/91 (13%) of items relating to the nasal and oral cavities and pharynx and larynx were rated as core/recommended (Table 4). For the nasal cavity, the only two (out of 16) items recommended for inclusion were the function of the nasal cavity and regions for olfaction and respiration. Items relating to the oral cavity that were recommended (3/23) were the mouth, tongue, and salivary glands. None of the six items relating to the paranasal sinuses were rated as core/recommended. For the pharynx, only 4/18 items (including the pharynx as a whole and its major subdivisions) were included as recommended. Similarly, only 3/28 items relating to the larynx were rated as recommended (including the larynx as a whole, the thyroid cartilage and glandular structures).

### 3.4 Brain

Of the 366 items in this category, 311 (85%) were considered core/recommended (Table 5). All 14 items relating to cerebral gray and white matter, and all 18 items relating to the functional areas of the cerebrum were included, with the majority being rated as core. Most items (39/44) relating to the lobes of the brain were rated as core/recommended, with the only items not included relating to certain gyri and sulci in the temporal and occipital lobes. Most (30/31) pathologies associated with the cerebral hemispheres were also included. Also considered core/recommended were all 14 items relating to the limbic system, and most items relating to the diencephalon (18/24), basal nuclei/ganglia (27/32), brainstem (38/41), and cerebellum (30/38), including all conceptual items and most items relating to pathologies associated with these structures.

For the meninges, all items except the cerebellomedullary, interpeduncular, and pontine cisterns were rated core/recommended, including the majority (7/9) of pathologies. All (18/18) ventricular system items were classified as core/recommended, as well as three out of the four pathologies associated with this system. In contrast, only 60% of items (29/49) relating to the blood supply of brain structures were considered core/recommended; however, all 12 pathologies relating to blood supply were included.

### 3.5 Cranial Nerves, Special Senses, and Neural Pathways

Of the 272 items in this category, 206 (76%) were classified as core/recommended (Table 6). With regard to the 79 cranial nerve items, approximately three‐quarters related to cranial nerve pathways and functions were rated core/recommended (40/52, 77%). The majority of items relating to the somatic plexuses (1/1), pathology (6/7), and clinical examination (14/19) of the cranial nerves were deemed core/recommended. Of the 65 items relating to special senses, the proportion rated core/recommended was higher for hearing and balance (28/36) compared to vision (8/17), and only one pathology item was included in each category (vision: diabetic retinopathy; hearing and balance: vertigo).

Almost all items were rated core/recommended for motor control (47/49) and sensation (45/46), including topics relating to anatomy, function, pathways, concepts, and pathology. All tracts/pathways and their function were rated core/recommended except the cuneocerebellar pathway, where the function, but not the tract, was rated core/recommended. With respect to the ANS, only 45% (15/33) of items were considered core/recommended, including less than half of the anatomy and function items (10/28), but all items relating to concepts and pathology (5/5) were included. Of note, while the nerve plexus item was rated core/recommended, none of the named plexuses (e.g., cardiac, esophageal, coeliac) were. For the peripheral nervous system and sympathetic nervous system, many of the named ganglion and splanchnic nerves were not included.

### 3.6 Open Comments

The panelists contributed 233 comments in Phase 2, which mostly comprised feedback and justification. In general, many of the feedback (*n* = 76) and justification (*n* = 93) comments related to which area of the curriculum was best suited for some of the items. This was particularly true of pathology items, with numerous comments relating to the suggestion that pathology would be taught in courses other than neuroanatomy (e.g., in neuropathology or pathophysiology courses). However, it was also acknowledged that it is useful for students to have an understanding of common pathologies to highlight anatomy and function as well as their clinical application, especially in relation to the brain. Similarly, some items could equally feature in biomechanics (e.g., temporomandibular joint function), musculoskeletal anatomy (e.g., muscles of the head and neck) or clinically focused (e.g., cranial nerve testing) curriculum. All eight of the suggested modifications were acted on, and two items that had been duplicated were removed from the list. Of the 57 suggested additions, 26 were actioned, 14 already existed in other areas of the topic list, and 17 were considered too detailed for a minimally competent physical therapy student. In Phase 3, 15 feedback or justification comments accompanied the topic ratings, focused again on the overlap of anatomy‐related content with other aspects of the curriculum, and whether knowledge was appropriate for a minimally competent student (or was deemed to be too advanced).

## 4 Discussion

This study used a modified Delphi approach to establish a detailed head, neck, and neuroanatomy syllabus containing topics related to anatomical structures, function, concepts, and clinically relevant pathologies, which may be adopted within physical therapy curriculum worldwide. Of the 1001 items in the final topic list, 675 (75%) were rated as core or recommended knowledge for a minimally competent physical therapy student. In terms of topics across the different categories, those relating to the central nervous system dominated the core/recommended items (general nervous system = 38/50, 75%; brain = 311/366, 85%; cranial nerves, special senses, and neural pathways = 206/272, 76%) compared to musculoskeletal and visceral structures of the head and neck (bones and muscles of the head and neck = 108/222, 49%; the nasal and oral cavities, pharynx and larynx = 12/91, 13%).

Physical therapy management is an integral component in the assessment and rehabilitation of neurological conditions, which affect more than 3 billion people worldwide (Garner et al. 2023; World Health Organization 2024; World Physiotherapy 2023). Our findings highlight the necessity for physical therapy students to have a detailed understanding of neuroanatomy, particularly of individual neuroanatomical structures as well as neural connectivity. Importantly, most items relating to concepts, which describe functions of individual structures and functional connections between structures, as well as pathologies, were retained in the core syllabus. The inclusion of these concepts demonstrates the perceived importance of functional and clinical understandings of neuroanatomy for physical therapy students. These may provide a basis for understanding neurological conditions and to help students link neuroanatomy to clinical patient populations—so they can better grasp the complex nature of the nervous system, which can be difficult to comprehend without considering its functions or understanding what happens when “something goes wrong.”

The complexity of the nervous system also closely aligns with the concept of “neurophobia,” a term used to describe the fear and anxiety associated with learning neurological topics. Neurophobia is prevalent across the basic sciences and health professions, particularly medicine (Flanagan et al. 2007; Hernando‐Requejo 2020; Javaid et al. 2018; Jozefowicz 1994). Recently, neurophobia has also been identified in a physical therapy context, with Turkish students perceiving neurology, including neuroanatomy, to be the most challenging discipline across their program (Abasiyanik et al. 2024). However, in an attempt to combat neurophobia, focusing on the clinical application of neuroanatomy (e.g., pathology items) is known to be a useful way to both engage students and draw immediate relevance to future clinical practice (Hernando‐Requejo 2020; Sravanam et al. 2023).

In terms of content, the proportion of core/recommended head, neck, and neuroanatomy items (67%) is approximately 10% less than that of the musculoskeletal syllabus (77.5%) for physical therapy students, but the total number of items is considerably smaller (675 vs. 1699) (Woodley et al. 2023). In contrast, medical students were found to require a more comprehensive understanding of neuroanatomy, with a greater number of topics deemed core or recommended—555 (medicine) (Moxham et al. 2015) compared to approximately 420 (physical therapy). When considering the knowledge expected of newly qualified physical therapists, the smaller number of head, neck, and neuroanatomy items matches previous studies that show these areas are perceived to be less important by both students (Mattingly and Barnes 1994) and practicing clinicians (Latman and Lanier 2001). Similarly, in recent anatomical education publications, the proportion of recommended neuroanatomy learning objectives for entry‐level physical therapists is small in comparison to those related to musculoskeletal anatomy (Gangata et al. 2023; Pascoe and Rapport 2022). This is most marked in the study by Gangata et al. (2023) where 18 of the 182 anatomy learning outcomes are focused on neuroanatomy, although another seven learning outcomes do also address neurology (e.g., key principles and histology). The weighting of this content somewhat aligns with the emphasis on the musculoskeletal system and movement of the human body, which is one of the foundations of physical therapy (American Physical Therapy Association 2015). However, the musculoskeletal and nervous systems are not mutually exclusive but instead interact at the cellular, tissue, and macroscopic levels. Therefore, it is important that students understand the relationship between neuroanatomy and musculoskeletal anatomy, as well as how these systems interact with other systems (e.g., cardiovascular and pulmonary) and the relevant basic sciences (e.g., physiology, biomechanics, and neuroscience) (Carroll et al. 2022).

Head and neck anatomy is also viewed as a challenging topic for students to learn, often considered the second most difficult subject, behind neuroanatomy (Hall et al. 2018; Rehman et al. 2022). Our findings demonstrate that a reasonable base of knowledge is required for bones and muscles of the face (108/222 items core/recommended), but that the detailed anatomy of the oral and nasal cavities, or the pharynx and larynx (12/91 items core/recommended), is not considered highly relevant to physiotherapy clinical practice. These data concur with those of Gangata et al. (2023), who report a high rate of rejection of learning outcomes related to this region, with only two learning objectives specific to the head and neck (out of a total of 182) retained in their physical therapy syllabi. Anatomy of the head and neck is more relevant to the training of other health professionals, as indicated by a greater number of core/recommended head and neck items (*n* = 279) in the core syllabus for medical students (Tubbs et al. 2014), and the broad scope afforded by the 65 (of a total of 147) learning outcomes in the Anatomical Society's syllabus for undergraduate dental students (Matthan et al. 2020). As an example, a single outcome in the Anatomical Society's syllabus (e.g., learning outcome 49: “describe and identify the major foramina of the craniofacial skeleton”) would encompass multiple items contained within this proposed physical therapy syllabus (e.g., foramen magnum, foramen ovale, carotid canal etc.). This is not surprising, given that dentistry is founded in surgical sciences, requiring a detailed knowledge of head, and to a lesser extent neck, anatomy, and the links to relevant neuroanatomy (e.g., pain pathways) (McHanwell and Matthan 2020). Similarly, students preparing to practice medicine require an understanding of head and neck anatomy that can be applied both within and between different fields of speciality, such as radiology, surgery, and anesthetics (Rehman et al. 2022; Tubbs et al. 2014).

The number of items relating to the cranial nerves (*n* = 79), special senses (*n* = 38), and neural pathways relevant to motor control (*n* = 47) and sensation (*n* = 45) that were deemed core or recommended for physical therapy education should be expected, given these components of neuroanatomy are an essential part of everyday clinical practice. For example, a physical therapist may be the initial point of contact for patients presenting with neck pain or dysfunction, orofacial pain, and headaches, all of which could be symptoms of more severe underlying cranial nerve lesions (Finsterer and Grisold 2015; Taylor et al. 2021). Similarly, as many aspects of physical therapy focus on balance and the vestibular system across different subdisciplines, this likely explains the greater emphasis on the number of syllabus items relating to the special senses of hearing and balance (78%) compared to vision (47%). When compared to the medical syllabus (Moxham et al. 2015), a higher proportion of motor control and sensation items are considered essential for physical therapy, reflecting the relative clinical importance placed on these across the profession. The emphasis on motor control and sensation aligns with the study by Gangata et al. (2023) where half of the learning outcomes for neuroanatomy are focused on these aspects.

In contrast, although the anatomy and function of the ANS were deemed important to include in a preregistration physical therapy syllabus, the granular detail was not. Compared to the medical syllabus which has a proposed 44 core or recommended ANS items (Moxham et al. 2015), only six items (all which were general overview concepts) were core, and another nine recommended, in the current physical therapy syllabus. Although physical therapists may see patients with autonomic dysfunction (e.g., associated with spinal cord injury) (American College of Surgeons 2022; Wadsworth et al. 2021) some aspects of this topic area may be regarded as more specialized, and the symptoms may be best suited to pharmacological management (e.g., blood pressure medication) and therefore out of the scope of physical therapy practice.

As identified by members of our Delphi panel and in previous studies that have focused on the development of anatomy syllabi (Gelb et al. 2021; Moxham et al. 2015; Tubbs et al. 2014), it is likely that some items of this syllabus may be better incorporated into other areas of the curriculum, such as in clinically based subjects teaching topics like kinesiology, biomechanics, and pathology. However, we maintain that it is important to present this detailed list of identified items, but we emphasize that this should be used flexibly, particularly in relation to when and where each component is taught within a physical therapy program. Further, this study provides a guide for core anatomy content that can be mapped to learning outcomes for preregistration physical therapy programs to ensure they align with the expected core competencies and relevant professional accreditation requirements.

### 4.1 Limitations

This study adopted a purposive sampling approach, including snowball sampling, with the aim of obtaining viewpoints from a wide range of anatomists and clinicians as panelists with world‐wide representation. Although the composition of this panel covered most of the continents, we had the greatest representation from North America, Canada, and Europe, with relatively few panelists from regions such as South America, Africa, and Asia. Therefore, not all nationalities or perspectives are represented in these data. While the size of our Delphi panel is considered optimal, we did not use a predefined criterion to explicitly define how experts were chosen as panel members (Nasa et al. 2021), potentially introducing selection bias. Most items in the online survey were in a format whereby they were able to be rated. However, there were a few exceptions, with one being the temporomandibular joint, which was incorporated as a subheading rather than a topic item that was available to the panel for consideration. Presenting all headings and subheadings for rating may have been useful in better understanding their relevance and importance in terms of core or recommended topics in the neuroanatomy, head and neck syllabus.

## 5 Conclusion

This study presents an international core anatomy syllabus tailored to physical therapy education, comprising 675 core and recommended structures and concepts related to the head, neck, and neuroanatomy. A large focus of this IFAA syllabus is on the central nervous system, with less emphasis placed on musculoskeletal and visceral structures of the head and neck. This reflects what physical therapists usually encounter in clinical practice, particularly when assessing and managing people with neurological conditions. This detailed topic list aims to guide anatomy educators and physical therapy students, and will also be presented for international consideration as per the IFAA modified Delphi approach.

## Supporting information


**Data S1:** Supporting Information.

## Data Availability

The data that support the findings of this study are available from the corresponding author upon reasonable request.
