# Supplementary material for: A Core Head, Neck, and Neuroanatomy Syllabus for Physical Therapy Student Education
Source: Clin Anat. 2025 Aug 11;39(4):436–60. doi: 10.1002/ca.70016 (PMC13060005; doi:10.1002/ca.70016)
Supplement: Supplementary file 1 — Data S1: Supporting Information. [file CA-39-436-s001.docx]

**Supplementary tables**

| **KEY** | |
| --- | --- |
| CORE | > 60% |
| RECOMMENDED | 30% to 59% |
| NOT RECOMMENDED | 20% to 29% |
| NOT CORE | < 20% |

**Table S1. General Nervous System**

| **Topic** | **Essential** | **Important** | **Acceptable** | **Not Required** |
| --- | --- | --- | --- | --- |
| **Concepts** | | | | |
| Central nervous system (CNS) | 98% | 2% | 0% | 0% |
| Peripheral nervous system (PNS) | 98% | 2% | 0% | 0% |
| Anterior (ventral), rostral, caudal and posterior (dorsal) terminology | 64% | 25% | 11% | 0% |
| Myelination | 62% | 27% | 11% | 0% |
| Decussation | 62% | 36% | 2% | 0% |
| Neuronal regeneration | 60% | 27% | 11% | 2% |
| Neuronal aging | 40% | 45% | 11% | 4% |
| Neuronal plasticity | 64% | 25% | 7% | 4% |
| Consciousness | 47% | 20% | 33% | 0% |
| **Connective Tissue** | | | | |
| Endoneurium | 36% | 29% | 33% | 2% |
| Perineurium | 40% | 25% | 33% | 2% |
| Epineurium | 42% | 22% | 34% | 2% |
| **Development of the Nervous System** | | | | |
| Development of the brain from neurulation to formation of vesicles | 27% | 35% | 29% | 9% |
| Development of the medulla oblongata (myelencephalon) | 13% | 33% | 45% | 9% |
| Development of the pons (anterior metencephalon) | 13% | 29% | 47% | 11% |
| Development of the cerebellum (posterior metencephalon) | 15% | 27% | 49% | 9% |
| Development of the midbrain (mesencephalon) | 16% | 29% | 44% | 11% |
| Development of the cerebrum (prosencephalon) | 20% | 27% | 44% | 9% |
| Development of the diencephalon | 13% | 31% | 45% | 11% |
| Development of the telencephalon | 18% | 24% | 49% | 9% |
| Development of the cerebral cortex | 24% | 31% | 36% | 9% |
| **Nerve Fiber Types** | | | | |
| General somatic afferent fibers (i.e., afferent fibers for general sensation from somatic structures) | 82% | 16% | 2% | 0% |
| General visceral afferent fibers (i.e., afferent fibers for general sensation from visceral structures) | 51% | 31% | 18% | 0% |
| Special somatic afferent fibers (i.e., afferent fibers for special senses of sight, hearing and balance) | 73% | 20% | 7% | 0% |
| Special visceral afferent fibers (i.e., afferent fibers for special senses of taste and smell) | 36% | 27% | 33% | 4% |
| General somatic efferent fibers (i.e., efferent fibers that innervate skeletal muscle derived from somites) | 85% | 13% | 2% | 0% |
| Sympathetic general visceral efferent fibers (i.e., efferent fibers for sympathetic stimulation of relevant visceral structures) | 38% | 46% | 16% | 0% |
| Pre-ganglionic sympathetic general visceral efferent fibers | 33% | 40% | 25% | 2% |
| Post-ganglionic sympathetic general visceral efferent fibers | 34% | 42% | 22% | 2% |
| Parasympathetic general visceral efferent fibers (i.e., efferent fibers for parasympathetic stimulation of relevant visceral structures) | 44% | 40% | 16% | 0% |
| Pre-ganglionic parasympathetic general visceral efferent fibers | 31% | 38% | 29% | 2% |
| Post-ganglionic parasympathetic general visceral efferent fibers | 31% | 38% | 29% | 2% |
| Special visceral efferent/pharyngeal efferent fibers (i.e., efferent fibers that innervate skeletal muscle derived from pharyngeal arches) | 42% | 29% | 27% | 2% |
| Interneurons | 51% | 27% | 15% | 7% |
| **Pathology** | | | | |
| Multiple sclerosis | 71% | 18% | 11% | 0% |
| Guillain-Barre syndrome | 51% | 31% | 13% | 5% |
| Neuropraxia | 53% | 22% | 25% | 0% |
| Axonotmesis | 53% | 22% | 25% | 0% |
| Neurotmesis | 51% | 27% | 22% | 0% |
| Spina bifida | 47% | 27% | 22% | 4% |
| Meningocele | 36% | 24% | 36% | 4% |
| Anencephaly | 22% | 29% | 38% | 11% |
| Megalencephaly | 20% | 29% | 42% | 9% |
| Cerebral palsy | 71% | 16% | 13% | 0% |
| Myasthenia gravis | 47% | 33% | 16% | 4% |
| Spinal cord injury/syndrome | 75% | 20% | 2% | 3% |
| Vascular injury/condition | 50% | 40% | 5% | 5% |
| Nervous system tumour | 21% | 29% | 45% | 5% |
| Differentiate between an upper motor neuron and lower motor neuron lesion | 78% | 17% | 5% | 0% |
| Common gait anomalies associated with nervous system | 55% | 33% | 10% | 2% |

**Table S2. Bones and Muscles of the Head and Neck**

| **Bones of the Skull – Neurocranium** | **Essential** | **Important** | **Acceptable** | **Not Required** |
| --- | --- | --- | --- | --- |
| ***Frontal bone*** | 69% | 15% | 9% | 7% |
| Superciliary arch | 9% | 16% | 33% | 42% |
| Supraorbital notch | 20% | 22% | 29% | 29% |
| Frontal crest | 14% | 22% | 31% | 33% |
| Orbital cavity | 38% | 31% | 18% | 13% |
| ***Parietal bone*** | 71% | 15% | 7% | 7% |
| ***Temporal bone*** | 69% | 17% | 7% | 7% |
| Squamous part of temporal bone | 20% | 49% | 20% | 11% |
| Petrous part of temporal bone | 25% | 42% | 20% | 13% |
| Superior temporal line | 11% | 22% | 42% | 25% |
| Inferior temporal line | 11% | 22% | 42% | 25% |
| Internal acoustic meatus | 36% | 36% | 22% | 6% |
| External acoustic meatus | 38% | 33% | 22% | 7% |
| Zygomatic process of temporal bone | 44% | 29% | 18% | 9% |
| Mastoid process | 51% | 31% | 13% | 5% |
| Styloid process of temporal bone | 38% | 40% | 13% | 9% |
| Articular tubercle | 36% | 31% | 24% | 9% |
| Mandibular fossa | 51% | 27% | 13% | 9% |
| Stylomastoid foramen | 34% | 42% | 13% | 11% |
| Jugular foramen | 41% | 39% | 11% | 9% |
| Groove for sigmoid sinus | 22% | 29% | 31% | 18% |
| Temporal fossa | 24% | 36% | 24% | 16% |
| Infratemporal fossa | 20% | 33% | 31% | 16% |
| ***Occipital bone*** | 69% | 15% | 9% | 7% |
| Foramen magnum | 60% | 33% | 5% | 2% |
| External occipital protuberance | 47% | 31% | 18% | 4% |
| Internal occipital protuberance | 18% | 33% | 36% | 13% |
| Superior nuchal line | 40% | 35% | 18% | 7% |
| Inferior nuchal line | 35% | 38% | 20% | 7% |
| Internal occipital crest | 11% | 23% | 35% | 31% |
| Groove for transverse sinus | 22% | 24% | 36% | 18% |
| Occipital condyle | 51% | 27% | 15% | 7% |
| Posterior condylar canal | 11% | 25% | 33% | 31% |
| Foramen lacerum | 24% | 27% | 29% | 20% |
| Hypoglossal canal | 33% | 35% | 16% | 16% |
| ***Sphenoid bone*** | 60% | 22% | 11% | 7% |
| Body of sphenoid bone | 36% | 40% | 15% | 9% |
| Greater wing | 33% | 40% | 18% | 9% |
| Lesser wing | 31% | 40% | 20% | 9% |
| Spine of sphenoid bone | 13% | 20% | 42% | 25% |
| Optic foramen | 36% | 33% | 20% | 11% |
| Optic canal | 36% | 40% | 13% | 11% |
| Pituitary fossa | 36% | 36% | 15% | 13% |
| Foramen rotundum | 36% | 33% | 18% | 13% |
| Foramen ovale | 36% | 33% | 22% | 9% |
| Foramen spinosum | 25% | 42% | 20% | 13% |
| Carotid canal | 36% | 36% | 15% | 13% |
| Superior orbital fissure | 42% | 22% | 27% | 9% |
| Inferior orbital fissure | 33% | 25% | 33% | 9% |
| Medial pterygoid plate | 27% | 27% | 31% | 15% |
| Lateral pterygoid plate | 29% | 27% | 29% | 15% |
| Pterygoid hamulus | 13% | 18% | 42% | 27% |
| ***Ethmoid bone*** | 56% | 22% | 15% | 7% |
| Cribriform plate | 38% | 35% | 18% | 9% |
| Perpendicular plate of ethmoid bone | 16% | 35% | 29% | 20% |
| Bony superior nasal concha | 18% | 35% | 29% | 18% |
| Bony middle nasal concha | 18% | 33% | 31% | 18% |
| Crista galli | 23% | 27% | 36% | 14% |
| Inferior nasal concha bone | 22% | 36% | 29% | 13% |
| ***Cranial fossae*** | 60% | 22% | 11% | 7% |
| Anterior fossa | 47% | 33% | 11% | 9% |
| Middle fossa | 49% | 31% | 11% | 9% |
| Posterior fossa | 49% | 31% | 11% | 9% |
| Cerebellar fossa | 42% | 34% | 13% | 11% |
| **Neurocranium – Concepts** | | | | |
| Intramembranous ossification process | 33% | 24% | 31% | 12% |
| Function of skull bones (e.g., protection of brain) | 71% | 19% | 10% | 0% |
| Surface anatomy and palpation of the head and neck | 53% | 30% | 12% | 5% |
| **Neurocranium – Pathology** | | | | |
| Craniosynostosis | 12% | 26% | 41% | 21% |
| Cribra orbitalia | 5% | 12% | 38% | 45% |
| Porotic hyperostosis | 5% | 12% | 36% | 47% |
| Fracture at/near pterion | 36% | 26% | 24% | 14% |
| **Joints – Cranial Sutures** | | | | |
| Joint type: fibrous suture (dense fibrous connective tissue) | 62% | 29% | 7% | 2% |
| Movement: Little to no movement | 62% | 26% | 7% | 5% |
| Coronal suture | 40% | 38% | 17% | 5% |
| Sagittal suture | 40% | 38% | 17% | 5% |
| Lambdoid suture | 40% | 38% | 17% | 5% |
| Bregma | 31% | 36% | 21% | 12% |
| Pterion | 40% | 31% | 19% | 10% |
| Purpose of fontanelles | 47% | 36% | 12% | 5% |
| **Bones of the Skull – Facial Skeleton** | | | | |
| ***Maxilla*** | 74% | 12% | 9% | 5% |
| Infraorbital foramen | 29% | 36% | 26% | 9% |
| Nasal spine of maxilla | 17% | 19% | 47% | 17% |
| Nasolacrimal canal | 14% | 29% | 48% | 9% |
| Alveolar process | 12% | 33% | 38% | 17% |
| Incisive canals | 7% | 21% | 36% | 36% |
| Pterygopalatine fossa | 17% | 19% | 40% | 24% |
| Inferior alveolar margin | 9% | 17% | 43% | 31% |
| Maxillary tuberosity | 19% | 14% | 31% | 36% |
| Hard palate | 31% | 36% | 24% | 9% |
| ***Lacrimal*** | 40% | 31% | 17% | 12% |
| Lacrimal foramen | 9% | 24% | 36% | 31% |
| ***Zygomatic bone*** | 60% | 28% | 7% | 5% |
| Zygomatic arch | 43% | 38% | 14% | 5% |
| ***Mandible*** | 72% | 19% | 7% | 2% |
| Angle of mandible | 52% | 29% | 14% | 5% |
| Neck of mandible | 45% | 26% | 24% | 5% |
| Body of mandible | 48% | 33% | 14% | 5% |
| Ramus of mandible | 50% | 31% | 12% | 7% |
| Mylohyoid line | 21% | 17% | 48% | 14% |
| Sublingual fossa | 14% | 17% | 38% | 31% |
| Submandibular fossa | 14% | 14% | 46% | 26% |
| Digastric fossa | 17% | 17% | 40% | 26% |
| Genial (mental) spine | 14% | 19% | 36% | 31% |
| Mandibular foramen | 33% | 38% | 19% | 10% |
| Lingula of mandible | 17% | 17% | 47% | 19% |
| Mandibular symphysis | 17% | 9% | 48% | 26% |
| Oblique line of mandible | 17% | 12% | 33% | 38% |
| Mental foramen | 29% | 26% | 33% | 12% |
| Mandibular notch | 31% | 19% | 33% | 17% |
| Mandibular condyle | 50% | 33% | 10% | 7% |
| Coronoid process of mandible | 50% | 31% | 12% | 7% |
| Mental protuberance | 19% | 19% | 43% | 19% |
| ***Vomer*** | 36% | 21% | 29% | 14% |
| Nasal septum | 16% | 36% | 36% | 12% |
| ***Palatine bone*** | 43% | 19% | 26% | 12% |
| Greater palatine foramina | 12% | 10% | 45% | 33% |
| Lesser palatine foramina | 12% | 9% | 43% | 36% |
| Horizontal plate of palatine bone | 12% | 17% | 52% | 19% |
| Pyramidal process | 12% | 14% | 31% | 43% |
| ***Nasal bone*** | 45% | 26% | 22% | 7% |
| Nasal aperture | 24% | 26% | 31% | 19% |
| ***Hyoid bone*** | 55% | 31% | 9% | 5% |
| Body of hyoid bone | 19% | 38% | 33% | 10% |
| Greater horn of hyoid bone | 12% | 43% | 33% | 12% |
| Lesser horn of hyoid bone | 12% | 43% | 33% | 12% |
| **Joints – Temporomandibular Joint** | | | | |
| Movements: Retraction/retrusion, protraction/protrusion, depression and elevation and lateral deviation | 79% | 19% | 0% | 2% |
| Lateral temporomandibular ligament | 53% | 21% | 21% | 5% |
| Sphenomandibular ligament | 43% | 24% | 28% | 5% |
| Stylomandibular ligament | 43% | 24% | 26% | 7% |
| Articular disc of temporomandibular joint | 69% | 19% | 10% | 2% |
| Articular surfaces lined by fibrocartilage | 57% | 21% | 17% | 5% |
| Functional concept: Maximum stability of the temporomandibular joint is when the mouth is closed and teeth occluded | 55% | 26% | 17% | 2% |
| Functional concept: Superior and inferior joint cavities of the temporomandibular joint move in different ways to produce different movements e.g., superior produces gliding, inferior produces hinge-like movements | 60% | 26% | 12% | 2% |
| **Facial Skeleton – Pathology** | | | | |
| Temporomandibular joint disorders | 50% | 26% | 17% | 7% |
| Fractures of bones of the facial skeleton | 17% | 45% | 29% | 9% |
| Cleft palate/lip | 0% | 38% | 47% | 15% |
| **Muscles of the Head and Neck** | | | | |
| ***Primary muscles of mastication*** | 77% | 12% | 2% | 9% |
| Medial pterygoid muscle | 70% | 14% | 7% | 9% |
| Lateral pterygoid muscle | 70% | 14% | 7% | 9% |
| Masseter | 77% | 9% | 7% | 7% |
| Temporalis muscle | 77% | 11% | 5% | 7% |
| Secondary muscles of mastication | 23% | 36% | 30% | 11% |
| ***Muscles of facial expression*** | 38% | 44% | 9% | 9% |
| Occipitalis muscle | 41% | 34% | 16% | 9% |
| Frontalis muscle | 50% | 32% | 9% | 9% |
| Corrugator supercilii | 16% | 30% | 25% | 29% |
| Procerus muscle | 14% | 29% | 23% | 34% |
| Orbicularis oculi muscle | 43% | 34% | 14% | 9% |
| Levator palpebrae superioris | 27% | 46% | 18% | 9% |
| Nasalis muscle | 18% | 25% | 30% | 27% |
| Levator nasolabialis | 21% | 25% | 27% | 27% |
| Depressor septi nasi | 10% | 30% | 30% | 30% |
| Orbicularis oris muscle | 43% | 30% | 18% | 9% |
| Bucinator | 50% | 25% | 14% | 11% |
| Levator labii superioris | 21% | 27% | 25% | 27% |
| Depressor labii inferioris | 18% | 27% | 25% | 30% |
| Zygomaticus major muscle | 25% | 23% | 34% | 18% |
| Zygomaticus minor muscle | 21% | 25% | 36% | 18% |
| Levator anguli oris | 18% | 25% | 34% | 23% |
| Depressor anguli oris | 18% | 25% | 32% | 25% |
| Risorius muscle | 18% | 23% | 32% | 27% |
| Mentalis muscle | 16% | 23% | 34% | 27% |
| Platysma | 27% | 34% | 32% | 7% |
| ***Muscles of the eye*** | 48% | 29% | 16% | 7% |
| Sphincter pupillae | 27% | 43% | 21% | 9% |
| Dilator pupillae | 27% | 43% | 21% | 9% |
| ***Extraocular muscles*** | 52% | 30% | 14% | 4% |
| Superior oblique muscle | 45% | 32% | 16% | 7% |
| Inferior oblique muscle | 45% | 32% | 16% | 7% |
| Superior rectus muscle | 43% | 34% | 16% | 7% |
| Inferior rectus muscle | 43% | 34% | 16% | 7% |
| Medial rectus muscle | 43% | 34% | 16% | 7% |
| Lateral rectus muscle | 45% | 32% | 16% | 7% |
| ***Intrinsic muscles of the tongue*** | 27% | 30% | 30% | 13% |
| Superior longitudinal lingual muscle | 4% | 14% | 48% | 34% |
| Verticalis linguae muscle | 4% | 14% | 48% | 34% |
| Transversus linguae muscle | 5% | 16% | 45% | 34% |
| Inferior longitudinal lingual muscle | 5% | 16% | 45% | 34% |
| ***Extrinsic muscles of the tongue*** | 27% | 32% | 32% | 9% |
| Palatoglossus muscle | 11% | 18% | 48% | 23% |
| Styloglossus muscle | 11% | 18% | 50% | 21% |
| Genioglossus muscle | 11% | 27% | 41% | 21% |
| Hyoglossus muscle | 9% | 21% | 52% | 18% |
| ***Muscles of the pharynx*** | 25% | 34% | 32% | 9% |
| Superior pharyngeal constrictor | 9% | 32% | 41% | 18% |
| Middle pharyngeal constrictor | 9% | 32% | 41% | 18% |
| Inferior pharyngeal constrictor | 9% | 32% | 41% | 18% |
| Stylopharyngeus muscle | 4% | 32% | 41% | 23% |
| Salpingopharyngeus muscle | 4% | 23% | 48% | 25% |
| Palatopharyngeus muscle | 4% | 23% | 48% | 25% |
| Tensor veli palatini | 7% | 18% | 45% | 30% |
| ***Intrinsic muscles of the larynx*** | 23% | 27% | 34% | 16% |
| Posterior cricoarytenoid muscle | 9% | 23% | 39% | 29% |
| Vocalis muscle | 5% | 25% | 41% | 29% |
| Lateral cricoarytenoid muscle | 4% | 18% | 39% | 39% |
| Oblique arytenoid muscle | 4% | 18% | 39% | 39% |
| Cricothyroid muscle | 9% | 23% | 34% | 34% |
| Thyroarytenoid muscle | 4% | 18% | 39% | 39% |
| ***Extrinsic muscles of the larynx*** | 18% | 32% | 34% | 16% |
| ***Suprahyoid muscles*** | 30% | 32% | 27% | 11% |
| Stylohyoid muscle | 14% | 27% | 43% | 16% |
| Mylohyoid muscle | 14% | 27% | 45% | 14% |
| Geniohyoid muscle | 14% | 25% | 48% | 13% |
| Digastric muscle | 14% | 29% | 43% | 14% |
| ***Infrahyoid muscles*** | 30% | 34% | 27% | 9% |
| Sternohyoid muscle | 11% | 30% | 45% | 14% |
| Sternothyroid muscle | 11% | 30% | 45% | 14% |
| Thyrohyoid muscle | 11% | 30% | 45% | 14% |
| Omohyoid muscle | 11% | 30% | 45% | 14% |
| Muscles of the ear | 11% | 34% | 41% | 14% |
| Tensor tympani | 18% | 36% | 34% | 12% |
| Stapedius muscle | 9% | 34% | 43% | 14% |
| **Muscles of the Head and Neck – Functional Concepts** | | | | |
| Muscles of mastication – allow for chewing and grinding motions | 73% | 22% | 0% | 5% |
| Muscles of facial expression – have a role in opening or closing mouth, eyes and nose | 71% | 22% | 5% | 2% |
| Muscles of facial expression – have a role in expression of emotions | 66% | 24% | 7% | 2% |
| Unilateral and bilateral muscle contractions produce different movements at the temporomandibular joint | 61% | 22% | 15% | 2% |
| Synergistic activity of the pterygoid muscles produces different movements at the temporomandibular joint | 56% | 20% | 20% | 4% |
| Voice – controlled by contractions of the laryngeal muscles | 39% | 37% | 22% | 2% |
| Swallowing – controlled by muscles of the pharynx and tongue | 44% | 37% | 19% | 0% |
| Mechanical digestion – muscles of the tongue contribute to this | 37% | 37% | 24% | 2% |
| Muscles of the eye allow for change in pupil size | 51% | 27% | 22% | 0% |

**Table S3. Nasal and Oral Cavities, Pharynx and Larynx**

| **Topic** | **Essential** | **Important** | **Acceptable** | **Not Required** |
| --- | --- | --- | --- | --- |
| **Nasal Cavity** | | | | |
| External nose | 20% | 46% | 22% | 12% |
| Anterior nares | 7% | 49% | 27% | 17% |
| Superior meatus | 7% | 30% | 39% | 24% |
| Middle meatus | 7% | 30% | 39% | 24% |
| Inferior meatus | 7% | 30% | 39% | 24% |
| Vestibule | 7% | 17% | 44% | 32% |
| Vibrissae | 2% | 20% | 34% | 44% |
| Sebaceous and sweat glands | 5% | 12% | 34% | 49% |
| Olfactory receptor cells | 17% | 39% | 22% | 22% |
| Paranasal sinuses | 29% | 42% | 12% | 17% |
| Nasolacrimal duct | 19% | 27% | 37% | 17% |
| **Nasal Cavity – Concepts** | | | | |
| Function of nasal cavity | 37% | 44% | 19% | 0% |
| Nasal septum | 5% | 28% | 60% | 7% |
| Regions for olfaction and respiration | 32% | 44% | 22% | 2% |
| Drainage of paranasal sinuses into nasal cavity | 24% | 39% | 27% | 10% |
| Endotracheal intubation | 25% | 23% | 35% | 17% |
| **Paranasal Sinuses** | | | | |
| Sinus of frontal bone | 27% | 37% | 24% | 12% |
| Sinus of maxilla | 29% | 37% | 22% | 12% |
| Sinus of ethmoid bone | 17% | 46% | 25% | 12% |
| Sinus of sphenoid bone | 22% | 42% | 24% | 12% |
| **Paranasal Sinuses – Pathology** | | | | |
| Acute sinusitis | 17% | 20% | 51% | 12% |
| Chronic sinusitis | 17% | 19% | 49% | 15% |
| **Pharynx** | | | | |
| ***Pharynx*** | 46% | 26% | 24% | 2% |
| ***Nasopharynx*** | 39% | 27% | 32% | 2% |
| Posterior nares | 7% | 22% | 39% | 32% |
| Pharyngeal isthmus | 7% | 17% | 39% | 37% |
| Pharyngeal tonsils | 10% | 34% | 39% | 17% |
| ***Oropharynx*** | 39% | 27% | 32% | 2% |
| Isthmus of fauces (Oropharyngeal isthmus) | 7% | 17% | 47% | 29% |
| Soft palate | 24% | 27% | 42% | 7% |
| Palatopharyngeal arch | 10% | 19% | 49% | 22% |
| Palatoglossal arch | 10% | 19% | 49% | 22% |
| Palatine tonsil | 14% | 22% | 54% | 10% |
| Lingual tonsil | 10% | 17% | 58% | 15% |
| ***Laryngopharynx*** | 39% | 24% | 32% | 5% |
| Laryngeal inlet | 12% | 22% | 49% | 17% |
| **Pharynx – Pathology** | | | | |
| Pharyngitis | 7% | 25% | 39% | 29% |
| Adenoidal and tonsilar hypertrophy | 7% | 27% | 37% | 29% |
| Tonsilitis | 7% | 27% | 44% | 22% |
| Aspiration | 20% | 40% | 30% | 10% |
| **Oral Cavity** | | | | |
| ***Mouth*** | 44% | 32% | 19% | 5% |
| Lips | 29% | 29% | 29% | 13% |
| Internal surface of cheek | 7% | 20% | 44% | 29% |
| Gingiva | 7% | 17% | 44% | 32% |
| Oral vestibule | 7% | 22% | 42% | 29% |
| Oral cavity proper | 10% | 22% | 49% | 19% |
| Alveolar ridges | 7% | 12% | 39% | 42% |
| ***Tongue*** | 46% | 27% | 20% | 7% |
| Lingual papillae | 7% | 17% | 42% | 34% |
| Lingual frenulum | 5% | 22% | 29% | 44% |
| Filiform papillae | 5% | 12% | 42% | 41% |
| Taste buds | 5% | 29% | 39% | 27% |
| ***Salivary glands*** | 39% | 29% | 29% | 3% |
| Parotid gland | 24% | 44% | 27% | 5% |
| Parotid duct | 17% | 39% | 37% | 7% |
| Sublingual gland | 22% | 39% | 34% | 5% |
| Submandibular gland | 22% | 39% | 34% | 5% |
| **Oral Cavity – Pathology** | | | | |
| Oral cavity lesions | 5% | 15% | 29% | 51% |
| Oral ulcers | 5% | 10% | 32% | 53% |
| Gingivitis | 5% | 10% | 34% | 51% |
| Hand, foot and mouth disease | 5% | 10% | 39% | 46% |
| Canker sores | 0% | 10% | 36% | 54% |
| Herpes virus | 7% | 12% | 49% | 32% |
| **Larynx** | | | | |
| ***Larynx*** | 46% | 34% | 17% | 3% |
| Thyroid cartilage | 32% | 36% | 27% | 5% |
| Superior thyroid notch | 12% | 20% | 49% | 19% |
| Laryngeal prominence | 17% | 34% | 39% | 10% |
| Lamina of thyroid cartilage | 7% | 15% | 59% | 19% |
| Thyrohyoid membrane | 7% | 22% | 54% | 17% |
| Cricoid cartilage | 27% | 29% | 32% | 12% |
| Glottis | 22% | 41% | 27% | 10% |
| Supraglottic cavity | 7% | 17% | 47% | 29% |
| Infraglottic cavity | 7% | 17% | 47% | 29% |
| Arytenoid cartilage | 15% | 27% | 36% | 22% |
| True vocal folds | 15% | 39% | 34% | 12% |
| Vocal ligaments | 12% | 32% | 39% | 17% |
| Rima glottidis | 7% | 32% | 39% | 22% |
| Epiglottis | 29% | 37% | 27% | 7% |
| Recurrent laryngeal nerve | 17% | 44% | 32% | 7% |
| ***Joints*** | 24% | 25% | 34% | 17% |
| Cricoarytenoid joint | 10% | 22% | 44% | 24% |
| Cricothyroid joint | 7% | 24% | 42% | 27% |
| ***Glandular structures*** | 34% | 39% | 27% | 0% |
| Thyroid gland | 12% | 40% | 38% | 10% |
| Parathyroid glands | 24% | 49% | 27% | 0% |
| **Larynx – Pathology** | | | | |
| Vocal cord paralysis | 15% | 39% | 29% | 17% |
| Adenomas (thyroid and parathyroid) | 5% | 24% | 39% | 32% |
| Supraglottitis | 5% | 17% | 37% | 41% |
| Hashimoto thyroiditis | 7% | 12% | 39% | 42% |
| Goitre | 10% | 22% | 39% | 29% |
| Laryngitis | 7% | 22% | 42% | 29% |

**Table S4. Brain**

| **Topic** | **Essential** | **Important** | **Acceptable** | **Not Required** |
| --- | --- | --- | --- | --- |
| **Cerebrum** | | | | |
| ***Grey matter*** | 91% | 7% | 2% | 0% |
| Cerebral cortex | 89% | 7% | 4% | 0% |
| Layers of the cerebral cortex | 39% | 27% | 23% | 11% |
| ***White matter*** | 89% | 9% | 2% | 0% |
| Commissural fibers | 66% | 25% | 7% | 2% |
| Association fibers | 62% | 27% | 9% | 2% |
| Projection fibers | 66% | 23% | 9% | 2% |
| Corpus callosum | 73% | 25% | 2% | 0% |
| Rostrum of corpus callosum | 41% | 18% | 27% | 14% |
| Genu of corpus callosum | 39% | 18% | 29% | 14% |
| Body of corpus callosum | 41% | 20% | 25% | 14% |
| Splenium of corpus callosum | 36% | 20% | 30% | 14% |
| Septum pellucidum | 32% | 27% | 30% | 11% |
| Corona radiata | 55% | 29% | 14% | 2% |
| ***Major sulci and fissures*** | 84% | 11% | 5% | 0% |
| Central sulcus | 84% | 12% | 2% | 2% |
| Longitudinal cerebral fissure | 78% | 18% | 2% | 2% |
| Transverse cerebral fissure | 57% | 27% | 11% | 5% |
| Lateral sulcus | 77% | 14% | 7% | 2% |
| Parieto-occipital sulcus | 64% | 25% | 9% | 2% |
| ***Frontal lobe*** | 91% | 5% | 4% | 0% |
| Primary functions: Voluntary motor function, language, planning, mood, smell and social judgement, personality, intellect and complex learning abilities | 89% | 9% | 2% | 0% |
| Precentral sulcus | 59% | 23% | 11% | 7% |
| Precentral gyrus | 75% | 18% | 5% | 2% |
| Superior frontal sulcus | 34% | 20% | 32% | 14% |
| Inferior frontal sulcus | 32% | 23% | 29% | 16% |
| Superior frontal gyrus | 39% | 25% | 23% | 13% |
| Middle frontal gyrus | 39% | 23% | 25% | 13% |
| Inferior frontal gyrus | 41% | 23% | 23% | 13% |
| Opercular part of inferior frontal gyrus | 32% | 23% | 29% | 16% |
| Triangular part of inferior frontal gyrus | 30% | 25% | 25% | 20% |
| Orbital part of inferior frontal gyrus | 30% | 25% | 27% | 18% |
| ***Parietal lobe*** | 84% | 7% | 9% | 0% |
| Primary functions: Somatosensory perception and integration of sensory information | 86% | 12% | 2% | 0% |
| Postcentral sulcus | 66% | 14% | 16% | 4% |
| Postcentral gyrus | 82% | 11% | 5% | 2% |
| Intraparietal sulcus | 34% | 21% | 34% | 11% |
| Superior parietal lobule | 34% | 20% | 32% | 14% |
| Inferior parietal lobule | 34% | 20% | 30% | 16% |
| Supramarginal gyrus | 41% | 18% | 20% | 21% |
| Angular gyrus | 41% | 18% | 23% | 18% |
| ***Temporal lobe*** | 93% | 2% | 5% | 0% |
| Primary functions: Hearing, smell, learning, memory, fear and emotion | 86% | 12% | 2% | 0% |
| Transverse temporal gyri | 39% | 32% | 18% | 11% |
| Superior temporal gyrus | 34% | 36% | 21% | 9% |
| Middle temporal gyrus | 27% | 39% | 23% | 11% |
| Inferior temporal gyrus | 27% | 41% | 21% | 11% |
| Superior temporal sulcus | 27% | 27% | 32% | 14% |
| Inferior temporal sulcus | 27% | 25% | 34% | 14% |
| ***Occipital lobe*** | 86% | 9% | 5% | 0% |
| Primary function: Vision | 86% | 14% | 0% | 0% |
| Pre-occipital notch | 34% | 18% | 34% | 14% |
| Calcarine sulcus | 55% | 27% | 9% | 9% |
| Cuneus | 30% | 25% | 29% | 16% |
| Occipitotemporal (fusiform) gyri | 15% | 23% | 47% | 15% |
| Lingual gyrus | 30% | 27% | 29% | 14% |
| ***Insular lobe*** | 73% | 23% | 4% | 0% |
| Primary functions: Play a role in avoidance learning, decision-making, emotions and possibly addiction | 68% | 30% | 2% | 0% |
| **Cerebrum – Functional Areas** | | | | |
| ***Primary functional areas*** | 93% | 5% | 2% | 0% |
| Primary motor cortex | 91% | 7% | 0% | 2% |
| Primary somatosensory cortex | 91% | 5% | 2% | 2% |
| Primary auditory area | 87% | 9% | 2% | 2% |
| Primary visual cortex | 89% | 9% | 0% | 2% |
| ***Secondary functional areas*** | 82% | 11% | 5% | 2% |
| Premotor cortex | 84% | 7% | 5% | 4% |
| Supplementary motor areas | 80% | 9% | 7% | 4% |
| Broca area | 89% | 9% | 0% | 2% |
| Function of Broca area: Centre for expressive (motor) speech | 86% | 12% | 0% | 2% |
| Secondary somatosensory cortex | 57% | 18% | 20% | 5% |
| Secondary auditory areas | 48% | 25% | 23% | 4% |
| Secondary visual areas | 50% | 25% | 20% | 5% |
| ***Association areas*** | 73% | 18% | 5% | 4% |
| Association somatosensory areas | 66% | 21% | 11% | 2% |
| Wernicke area | 86% | 9% | 5% | 0% |
| Function of Wernicke area: Permits comprehension of spoken and written language and creates plans for formulation of speech | 89% | 7% | 4% | 0% |
| Prefrontal cortex | 82% | 11% | 5% | 2% |
| **Cerebrum – Pathology** | | | | |
| Lesions of the primary visual cortex | 57% | 23% | 18% | 2% |
| Lesions of the secondary visual area | 34% | 23% | 32% | 11% |
| Lesions of the primary auditory area | 48% | 25% | 20% | 7% |
| Lesions of the secondary auditory area | 34% | 18% | 34% | 14% |
| Lesions of the prefrontal cortex | 62% | 18% | 18% | 2% |
| Lesions of the primary somatosensory cortex | 68% | 21% | 9% | 2% |
| Lesions of the secondary somatosensory cortex | 43% | 21% | 27% | 9% |
| Lesions of the association somatosensory area | 43% | 18% | 32% | 7% |
| Lesions of the primary motor cortex | 82% | 11% | 7% | 0% |
| Lesions of the supplementary motor area | 57% | 20% | 21% | 2% |
| Lesions of the premotor cortex | 59% | 23% | 16% | 2% |
| Epilepsy | 34% | 34% | 30% | 2% |
| Aphasia (non-fluent) – lesion of Broca area | 52% | 30% | 18% | 0% |
| Aphasia (fluent) – lesion of Wernicke area | 52% | 30% | 18% | 0% |
| Apraxia | 59% | 20% | 21% | 0% |
| Astereognosis | 48% | 20% | 27% | 5% |
| Neglect syndrome (spatial neglect) | 62% | 9% | 27% | 2% |
| Contralateral homonymous hemianopia | 57% | 14% | 25% | 4% |
| Headaches caused by cerebral tumours | 27% | 30% | 36% | 7% |
| Dysarthria | 50% | 25% | 23% | 2% |
| Tonic spasm | 39% | 23% | 27% | 11% |
| Dysphagia | 45% | 32% | 18% | 5% |
| Alzheimer’s disease | 55% | 29% | 14% | 2% |
| Dementias | 50% | 32% | 14% | 4% |
| Brain tumours | 48% | 29% | 23% | 0% |
| Cerebral oedema | 52% | 32% | 16% | 0% |
| Traumatic brain injury | 55% | 35% | 8% | 2% |
| Concussions | 59% | 21% | 18% | 2% |
| Amyotrophic lateral sclerosis | 48% | 27% | 23% | 2% |
| Migraines | 36% | 21% | 36% | 7% |
| Headaches | 41% | 23% | 29% | 7% |
| **Diencephalon** | | | | |
| ***Thalamus*** | 89% | 9% | 2% | 0% |
| Function of the thalamus | 82% | 16% | 2% | 0% |
| Nuclei of the thalamus | 32% | 41% | 18% | 9% |
| Pulvinar | 14% | 27% | 43% | 16% |
| Stria medullaris thalami | 11% | 27% | 39% | 23% |
| Medial and lateral geniculate bodies | 34% | 36% | 21% | 9% |
| ***Hypothalamus*** | 80% | 18% | 2% | 0% |
| Function of the hypothalamus | 75% | 23% | 2% | 0% |
| Mammillary body | 32% | 41% | 20% | 7% |
| Nuclei of the hypothalamus | 12% | 34% | 36% | 18% |
| Optic chiasm | 59% | 30% | 7% | 4% |
| ***Epithalamus*** | 27% | 32% | 30% | 11% |
| Habenular complex | 12% | 27% | 34% | 27% |
| ***Pineal gland*** | 50% | 25% | 18% | 7% |
| Function of the pineal gland | 45% | 25% | 23% | 7% |
| ***Metathalamus*** | 13% | 32% | 32% | 23% |
| ***Subthalamus*** | 45% | 32% | 18% | 5% |
| Subthalamic nuclei | 41% | 30% | 18% | 11% |
| ***Pituitary gland*** | 57% | 34% | 9% | 0% |
| Adenohypophysis | 30% | 29% | 32% | 9% |
| Neurohypophysis | 30% | 29% | 32% | 9% |
| Neuroendocrine function | 36% | 39% | 23% | 2% |
| **Diencephalon – Concepts** | | | | |
| Arrangements and connections of the diencephalon | 46% | 41% | 11% | 2% |
| Function–Sensory integration | 50% | 33% | 17% | 0% |
| **Basal Nuclei/Ganglia** | | | | |
| ***Caudate nucleus*** | 70% | 23% | 5% | 2% |
| Head of caudate nucleus | 32% | 39% | 25% | 4% |
| Body of caudate nucleus | 32% | 39% | 25% | 4% |
| Tail of caudate nucleus | 32% | 36% | 27% | 5% |
| ***Lentiform nucleus*** | 68% | 25% | 5% | 2% |
| Putamen | 61% | 23% | 11% | 5% |
| Globus pallidus | 61% | 23% | 11% | 5% |
| Medial segment of globus pallidus | 25% | 45% | 23% | 7% |
| Lateral segment of globus pallidus | 25% | 43% | 25% | 7% |
| ***Substantia nigra*** | 75% | 18% | 7% | 0% |
| ***Internal capsule*** | 77% | 18% | 5% | 0% |
| Functions of the internal capsule | 73% | 25% | 2% | 0% |
| Genu of internal capsule | 48% | 16% | 32% | 4% |
| Posterior limb of internal capsule | 45% | 18% | 32% | 5% |
| Anterior limb of internal capsule | 43% | 20% | 32% | 5% |
| External capsule | 25% | 21% | 43% | 11% |
| Claustrum | 25% | 20% | 46% | 9% |
| Extreme capsule | 23% | 20% | 46% | 11% |
| **Basal Nuclei/Ganglia – Concepts** | | | | |
| Functions of the basal nuclei | 89% | 9% | 2% | 0% |
| Circuits between the basal nuclei, cerebral cortex and cerebellum | 66% | 27% | 7% | 0% |
| Input nuclei of the basal nuclei | 48% | 27% | 18% | 7% |
| Intrinsic nuclei of the basal nuclei | 45% | 23% | 23% | 9% |
| Output nuclei of the basal nuclei | 48% | 25% | 18% | 9% |
| **Basal Nuclei/Ganglia – Concepts** | | | | |
| Parkinson disease | 80% | 11% | 9% | 0% |
| Huntington disease | 57% | 27% | 16% | 0% |
| Hypokinesia | 59% | 20% | 16% | 5% |
| Hyperkinesia | 57% | 23% | 16% | 4% |
| Chorea | 61% | 14% | 23% | 2% |
| Athetosis | 55% | 20% | 20% | 5% |
| Hemiballismus | 52% | 23% | 20% | 5% |
| Tic | 41% | 34% | 16% | 9% |
| Resting tremor | 59% | 16% | 20% | 5% |
| **Limbic System** | | | | |
| ***Hippocampus*** | 77% | 14% | 9% | 0% |
| Function: Associated with formation of long-term memories | 77% | 12% | 9% | 2% |
| Hippocampal formation | 36% | 25% | 30% | 9% |
| ***Amygdalaloid body*** | 68% | 23% | 7% | 2% |
| Function: Signalling the cortex of motivational stimuli | 66% | 21% | 11% | 2% |
| Amygdaloid nuclei | 30% | 30% | 27% | 13% |
| ***Parahippocampal gyrus*** | 50% | 23% | 23% | 4% |
| Function: Associated with memory formation | 48% | 27% | 20% | 5% |
| ***Cingulate gyrus*** | 64% | 18% | 16% | 2% |
| Functions: Autonomic functions which regulate heart rate, blood pressure and processes such as cognition and attention | 57% | 25% | 16% | 2% |
| ***Fornix*** | 34% | 30% | 32% | 4% |
| Function: Carries signals from the hippocampus to the mammillary bodies | 30% | 36% | 27% | 7% |
| ***Dentate gyrus*** | 36% | 27% | 30% | 7% |
| Function: New memory formation and regulation of mood | 34% | 30% | 29% | 7% |
| **Limbic System – Concepts** | | | | |
| Connecting pathways of the limbic system | 41% | 30% | 27% | 2% |
| The role of the limbic system in emotion | 30% | 20% | 48% | 2% |
| **Limbic System – Pathology** | | | | |
| Disorders of the limbic system | 18% | 41% | 32% | 9% |
| **Brainstem** | | | | |
| ***Midbrain*** | 86% | 9% | 5% | 0% |
| Cerebral peduncle | 55% | 32% | 9% | 4% |
| Crus cerebri | 52% | 25% | 16% | 7% |
| Interpeduncular fossa | 41% | 20% | 32% | 7% |
| Superior colliculus | 55% | 25% | 16% | 4% |
| Inferior colliculus | 55% | 25% | 16% | 4% |
| Superior cerebellar peduncle | 55% | 18% | 23% | 4% |
| Red nucleus | 43% | 36% | 14% | 7% |
| Tectum of midbrain | 43% | 34% | 16% | 7% |
| ***Pons*** | 82% | 14% | 4% | 0% |
| Basilar part of pons | 39% | 32% | 20% | 9% |
| Basilar sulcus | 25% | 32% | 29% | 14% |
| Middle cerebellar peduncle | 50% | 30% | 20% | 0% |
| Main sensory nucleus of trigeminal nerve | 48% | 36% | 14% | 2% |
| ***Medulla oblongata*** | 91% | 5% | 4% | 0% |
| Pyramid of medulla oblongata | 82% | 11% | 5% | 2% |
| Inferior cerebellar peduncle | 55% | 27% | 18% | 0% |
| Anterior median fissure of medulla oblongata | 34% | 27% | 26% | 11% |
| Decussation of pyramids | 80% | 14% | 4% | 2% |
| Olive | 54% | 25% | 14% | 7% |
| Olivary nuclei | 43% | 34% | 16% | 7% |
| Vestibulocochlear nuclei | 48% | 30% | 18% | 4% |
| Gracile nucleus | 52% | 25% | 18% | 5% |
| Cuneate nucleus | 52% | 25% | 18% | 5% |
| Gracile fasciculus | 61% | 20% | 14% | 5% |
| Cuneate fasciculus | 61% | 20% | 14% | 5% |
| Nucleus ambiguus | 27% | 43% | 16% | 14% |
| Nucleus of solitary tract | 25% | 48% | 14% | 13% |
| Spinal nucleus of trigeminal nerve | 50% | 30% | 18% | 2% |
| ***Reticular formation*** | 71% | 25% | 2% | 2% |
| Reticular activating system nuclei | 33% | 40% | 25% | 2% |
| Functions of the reticular formation | 59% | 34% | 5% | 2% |
| **Brainstem – Concepts** | | | | |
| Associations of brainstem with descending and ascending tracts to and from spinal cord and cerebral cortex | 91% | 7% | 2% | 0% |
| Associations of brainstem with cerebellum | 91% | 7% | 2% | 0% |
| Tracts which originate in brainstem | 77% | 14% | 9% | 0% |
| **Brainstem – Pathology** | | | | |
| Lesions of the midbrain | 48% | 32% | 18% | 2% |
| Lesions of the pons | 43% | 34% | 21% | 2% |
| Lesions of the medulla oblongata | 46% | 34% | 18% | 2% |
| Lesions of the reticular formation | 43% | 30% | 20% | 7% |
| Loss of consciousness | 52% | 30% | 14% | 4% |
| Myoclonus | 34% | 21% | 36% | 9% |
| **Cerebellum** | | | | |
| ***Grey matter*** | 80% | 11% | 7% | 2% |
| Cerebellar cortex | 75% | 18% | 2% | 5% |
| Layers of cerebellar cortex | 43% | 23% | 25% | 9% |
| Fastigial nucleus | 23% | 39% | 25% | 13% |
| Emboliform nucleus | 23% | 39% | 25% | 13% |
| Globose nucleus | 23% | 39% | 25% | 13% |
| Dentate nucleus | 25% | 45% | 21% | 9% |
| ***Hemisphere of cerebellum*** | 77% | 18% | 2% | 3% |
| Lateral hemisphere | 66% | 25% | 5% | 4% |
| Function of lateral hemisphere: Integrative, projections to motor/pre-motor cortex | 71% | 25% | 2% | 2% |
| Vermis of cerebellum | 61% | 30% | 5% | 4% |
| Function of vermis: Responds to proprioceptive and somatosensory input | 61% | 30% | 7% | 2% |
| Folia of cerebellum | 20% | 25% | 41% | 14% |
| Lingula of cerebellum | 14% | 29% | 39% | 18% |
| ***Cerebellar lobes*** | 68% | 23% | 5% | 4% |
| Anterior lobe of cerebellum | 52% | 36% | 7% | 5% |
| Function of anterior lobe: Important in movement coordination | 66% | 23% | 9% | 2% |
| Middle lobe of cerebellum | 55% | 34% | 7% | 4% |
| Function of middle lobe: Important in movement coordination | 66% | 23% | 9% | 2% |
| Flocculonodular lobe | 52% | 36% | 7% | 5% |
| Function of flocculonodular lobe: Important for adjustments of posture to maintain balance and vestibular functions | 64% | 25% | 9% | 2% |
| Primary fissure | 34% | 34% | 30% | 2% |
| Flocculus | 39% | 32% | 27% | 2% |
| Nodule of vermis | 39% | 30% | 29% | 2% |
| Tonsil of cerebellum | 43% | 27% | 25% | 5% |
| ***White matter*** | 64% | 20% | 14% | 2% |
| Arbor vitae | 34% | 30% | 27% | 9% |
| Superior medullary velum | 18% | 27% | 34% | 21% |
| Inferior medullary velum | 18% | 27% | 34% | 21% |
| **Cerebellum – Pathology** | | | | |
| Ataxia | 75% | 20% | 5% | 0% |
| Dysmetria | 66% | 20% | 14% | 0% |
| Dysdiadochokinesia | 66% | 16% | 16% | 2% |
| Dyssynergia | 57% | 20% | 18% | 5% |
| Reflex disturbances | 57% | 25% | 14% | 4% |
| Postural and gait changes | 73% | 20% | 7% | 0% |
| Ocular movement disturbances | 59% | 16% | 25% | 0% |
| Disorders of speech | 45% | 20% | 32% | 2% |
| Intention tremor | 64% | 18% | 18% | 0% |
| **Meninges** | | | | |
| ***Dura*** | 86% | 7% | 7% | 0% |
| Periosteal cranial dura | 45% | 34% | 14% | 7% |
| Meningeal cranial dura | 46% | 36% | 11% | 7% |
| Layers are fused except for where they split to form venous sinuses | 48% | 34% | 11% | 7% |
| Falx cerebri | 55% | 30% | 11% | 4% |
| Falx cerebelli | 50% | 32% | 11% | 7% |
| Tentorium cerebelli | 55% | 32% | 9% | 4% |
| Subdural space | 64% | 25% | 9% | 2% |
| ***Arachnoid*** | 82% | 11% | 7% | 0% |
| Arachnoid granulations | 55% | 32% | 11% | 2% |
| Subarachnoid space | 68% | 27% | 2% | 3% |
| Cerebellomedullary cistern | 25% | 34% | 18% | 23% |
| Interpeduncular cistern | 25% | 32% | 20% | 23% |
| Pontine cistern | 25% | 32% | 20% | 23% |
| ***Pia*** | 80% | 11% | 5% | 4% |
| **Meninges – Pathology** | | | | |
| Subdural hematoma | 66% | 20% | 14% | 0% |
| Extradural hemorrhage | 64% | 18% | 18% | 0% |
| Subdural hemorrhage | 61% | 20% | 18% | 0% |
| Subarachnoid hemorrhage | 61% | 18% | 21% | 0% |
| Intracranial hemorrhage | 66% | 20% | 14% | 0% |
| Movements of brain in relation to meninges in head injury | 57% | 23% | 16% | 4% |
| Migraine headaches | 27% | 30% | 39% | 4% |
| Meningeal headaches | 27% | 30% | 36% | 7% |
| Meningitis | 34% | 32% | 30% | 4% |
| **Ventricular System** | | | | |
| ***Cerebrospinal fluid*** | 84% | 11% | 5% | 0% |
| Formation of cerebrospinal fluid | 55% | 32% | 9% | 4% |
| Circulation of cerebrospinal fluid | 57% | 32% | 7% | 4% |
| Absorption of cerebrospinal fluid | 52% | 32% | 11% | 5% |
| Choroid plexus | 50% | 25% | 23% | 2% |
| ***Ventricles*** | 77% | 18% | 5% | 0% |
| Lateral ventricle | 70% | 23% | 7% | 0% |
| Frontal horn | 32% | 36% | 27% | 5% |
| Body of lateral ventricle | 32% | 36% | 27% | 5% |
| Occipital horn | 30% | 36% | 30% | 4% |
| Temporal horn | 30% | 34% | 32% | 4% |
| Third ventricle | 70% | 21% | 9% | 0% |
| Interventricular foramen | 61% | 23% | 11% | 5% |
| Aqueduct of midbrain (cerebral aqueduct) | 66% | 23% | 9% | 2% |
| Fourth ventricle | 70% | 21% | 7% | 2% |
| Median aperture of fourth ventricle | 32% | 43% | 20% | 5% |
| Lateral aperture of fourth ventricle | 32% | 43% | 20% | 5% |
| Posterior cerebellomedullary cistern (cisterna magna) | 34% | 34% | 23% | 9% |
| **Ventricular System – Pathology** | | | | |
| Changes in intracranial pressure | 35% | 48% | 17% | 0% |
| Hydrocephalus | 55% | 29% | 16% | 0% |
| Raised cerebrospinal fluid pressure | 50% | 30% | 18% | 2% |
| Papilledema | 21% | 32% | 36% | 11% |
| **Blood Supply** | | | | |
| ***Arteries*** | 89% | 7% | 4% | 0% |
| Common carotid artery | 77% | 16% | 7% | 0% |
| Internal carotid artery | 84% | 11% | 5% | 0% |
| External carotid artery | 68% | 21% | 11% | 0% |
| Vertebrobasilar system | 82% | 14% | 4% | 0% |
| Anastomoses | 71% | 18% | 11% | 0% |
| Cerebral arterial circle (circle of Willis) | 91% | 5% | 4% | 0% |
| Anterior cerebral artery | 82% | 14% | 4% | 0% |
| Middle cerebral artery | 86% | 12% | 2% | 0% |
| Posterior cerebral artery | 84% | 14% | 2% | 0% |
| Anterior communicating artery | 75% | 20% | 5% | 0% |
| Posterior communicating artery | 75% | 18% | 7% | 0% |
| Basilar artery | 77% | 21% | 2% | 0% |
| Anterior inferior cerebellar artery | 64% | 23% | 11% | 2% |
| Superior cerebellar artery | 62% | 18% | 18% | 2% |
| Posterior inferior cerebellar artery | 64% | 20% | 14% | 2% |
| Maxillary artery | 23% | 29% | 23% | 25% |
| Superficial temporal artery | 20% | 32% | 23% | 25% |
| Ascending pharyngeal artery | 5% | 27% | 41% | 27% |
| Facial artery | 20% | 32% | 27% | 21% |
| Middle meningeal artery | 36% | 30% | 18% | 16% |
| Lingual artery | 7% | 27% | 34% | 32% |
| Ophthalmic artery | 18% | 30% | 34% | 18% |
| Central retinal artery | 14% | 25% | 41% | 20% |
| Superior thyroid artery | 7% | 18% | 43% | 32% |
| Inferior thyroid artery | 7% | 18% | 43% | 32% |
| Occipital artery | 13% | 16% | 39% | 32% |
| ***Veins*** | 68% | 16% | 11% | 5% |
| Superior sagittal sinus | 57% | 25% | 14% | 4% |
| Inferior sagittal sinus | 50% | 27% | 18% | 5% |
| Occipital sinus | 34% | 32% | 23% | 11% |
| Transverse sinus | 48% | 29% | 16% | 7% |
| Straight sinus | 38% | 32% | 23% | 7% |
| Superior petrosal sinus | 16% | 23% | 43% | 18% |
| Inferior petrosal sinus | 16% | 23% | 43% | 18% |
| Cavernous sinus | 34% | 34% | 23% | 9% |
| Confluence of sinuses | 36% | 30% | 25% | 9% |
| Sigmoid sinus | 36% | 34% | 21% | 9% |
| Internal jugular vein | 54% | 23% | 16% | 7% |
| Superior ophthalmic vein | 11% | 25% | 43% | 21% |
| Facial vein | 9% | 36% | 37% | 18% |
| ***Lymphatics*** | 54% | 23% | 16% | 7% |
| Thoracic duct | 41% | 30% | 20% | 9% |
| Superficial cervical nodes | 20% | 30% | 34% | 16% |
| Deep cervical nodes | 21% | 27% | 34% | 18% |
| Submandibular nodes | 14% | 32% | 34% | 20% |
| Parotid nodes | 14% | 27% | 36% | 23% |
| Meningeal lymphatics | 9% | 30% | 36% | 25% |
| Lymphatic drainage | 10% | 43% | 32% | 15% |
| **Blood Supply – Pathology** | | | | |
| Cerebral ischemia | 77% | 14% | 9% | 0% |
| Cerebral infarction | 77% | 11% | 12% | 0% |
| Cerebral aneurysm | 66% | 20% | 14% | 0% |
| Congenital aneurysm | 36% | 32% | 21% | 11% |
| Postural hypotension | 54% | 16% | 23% | 7% |
| Hypotension | 57% | 16% | 23% | 4% |
| Hypertension | 61% | 12% | 25% | 2% |
| Diseases that alter blood pressure interrupting cerebral circulation | 50% | 18% | 27% | 5% |
| Ischemic stroke | 75% | 14% | 11% | 0% |
| Hemorrhagic stroke | 75% | 14% | 11% | 0% |
| Transient ischemic attack | 71% | 18% | 11% | 0% |
| Lymphadenopathy | 30% | 20% | 32% | 18% |

**Table S5. Cranial Nerves and Special Senses**

| **Topic** | **Essential** | **Important** | **Acceptable** | **Not Required** |
| --- | --- | --- | --- | --- |
| **Cranial Nerves** | | | | |
| ***Olfactory nerve (I)*** | 64% | 25% | 9% | 2% |
| Function: Special sensory – smell | 64% | 25% | 11% | 0% |
| Pathway: Olfactory ganglion to olfactory processing areas | 27% | 27% | 32% | 14% |
| Central connections of the olfactory nerve | 20% | 27% | 32% | 21% |
| Olfactory epithelium | 18% | 23% | 48% | 11% |
| Olfactory bulb | 27% | 21% | 41% | 11% |
| Olfactory foramina | 25% | 16% | 45% | 14% |
| ***Optic nerve (II)*** | 73% | 25% | 2% | 0% |
| Function: Special sensory – vision | 73% | 20% | 7% | 0% |
| Pathway: Ganglion cells of retina to visual processing areas | 52% | 23% | 18% | 7% |
| Central connections of the optic nerve | 45% | 27% | 14% | 14% |
| ***Oculomotor nerve (III)*** | 70% | 25% | 5% | 0% |
| Function: Motor to extraocular muscles and parasympathetic motor to control pupil | 68% | 23% | 9% | 0% |
| Pathway: Ventral midbrain at level of superior colliculus to site of action | 48% | 16% | 25% | 11% |
| ***Trochlear nerve (IV)*** | 68% | 21% | 9% | 2% |
| Function: Motor to superior oblique muscle | 66% | 18% | 11% | 5% |
| Pathway: Dorsal aspect of midbrain below inferior colliculus to site of action | 46% | 16% | 27% | 11% |
| ***Trigeminal nerve (V)*** | 82% | 18% | 0% | 0% |
| Function: Sensation from face and motor to muscles of mastication | 82% | 14% | 4% | 0% |
| Pathway: Trigeminal ganglion to junction of pons and middle cerebellar peduncle (sensory), same junction to site of action (motor) | 52% | 23% | 20% | 5% |
| Ophthalmic nerve – sensation from upper third of face | 66% | 25% | 9% | 0% |
| Maxillary neve – sensation from middle third of face | 66% | 23% | 11% | 0% |
| Mandibular nerve – sensation from lower third of face and somatic motor to muscles of mastication | 66% | 25% | 9% | 0% |
| ***Abducens nerve (VI)*** | 70% | 23% | 5% | 2% |
| Function: Motor to lateral rectus muscle | 68% | 20% | 7% | 5% |
| Pathway: Junction of pons and pyramid of medulla oblongata to site of action | 41% | 21% | 27% | 11% |
| ***Facial nerve (VII)*** | 86% | 14% | 0% | 0% |
| Function: Motor to muscles of facial expression, parasympathetic motor to submandibular and sublingual salivary and lacrimal glands; sensation from skin of auricle; and special sensory for taste from anterior 2/3rds of tongue | 86% | 14% | 0% | 0% |
| Pathway: Geniculate ganglion (sensory) to lateral edge of pontomedullary junction, same junction to site of action (motor) | 50% | 25% | 16% | 9% |
| Posterior auricular nerve | 23% | 34% | 27% | 16% |
| Temporal branches of facial nerve | 23% | 41% | 20% | 16% |
| Zygomatic branches of facial nerve | 23% | 41% | 20% | 16% |
| Buccal branches of facial nerve | 23% | 39% | 23% | 16% |
| Marginal mandibular branches of facial nerve | 23% | 38% | 23% | 16% |
| Cervical branch of facial nerve | 20% | 41% | 23% | 16% |
| ***Vestibulocochlear nerve (VIII)*** | 82% | 18% | 0% | 0% |
| Function: Special sensory for hearing and balance | 80% | 18% | 2% | 0% |
| Pathway: Vestibular/spiral ganglion to lateral edge of the pontomedullary junction | 45% | 23% | 18% | 14% |
| ***Glossopharyngeal nerve (IX)*** | 73% | 25% | 2% | 0% |
| Function: Motor to pharyngeal muscles for swallowing, parasympathetic motor to parotid gland; sensory from posterior 1/3rd of tongue, posterior auricle, tragus, soft palate, pharynx, tympanic membrane and cavity, pharyngo-tympanic tube, mastoid cells, carotid bodies and sinus; special sensory for taste from posterior 1/3rd of tongue) | 68% | 27% | 2% | 3% |
| Pathway: Medulla to sites of action (motor), ganglia for taste and carotid bodies and sinus to medulla (sensory) | 34% | 30% | 18% | 18% |
| ***Vagus nerve (X)*** | 86% | 14% | 0% | 0% |
| Function: Motor to muscles of the pharynx, larynx and soft palate; parasympathetic motor to smooth muscle of digestive and respiratory tracts and cardiac muscle; special sensory for taste from epiglottis and palate; and sensation from thoracic and abdominal viscera, carotid sinus and carotid and aortic bodies, auricle, external acoustic meatus, and dura mater of posterior cranial fossa | 82% | 16% | 2% | 0% |
| Pathway: Lateral medulla to sites of action (motor), ganglion of CNX to lateral medulla (sensory) | 48% | 23% | 22% | 7% |
| ***Accessory nerve (XI)*** | 84% | 14% | 2% | 0% |
| Function: Motor to larynx (cranial root) and sternocleidomastoid and trapezius (spinal root) | 82% | 14% | 4% | 0% |
| Pathway: Cranial from lateral medulla posterior to olives, spinal from supraspinal nucleus to sites of action | 45% | 23% | 21% | 11% |
| Cranial root of accessory nerve | 25% | 29% | 32% | 14% |
| Spinal root of accessory nerve | 36% | 25% | 30% | 9% |
| ***Hypoglossal nerve (XII)*** | 68% | 30% | 2% | 0% |
| Function: Motor to intrinsic and extrinsic muscles of the tongue (except palatoglossus – CN X) | 66% | 27% | 7% | 0% |
| Pathway: Rootlets between olive and pyramid of medulla oblongata to sites of action | 30% | 34% | 18% | 18% |
| **Cranial Nerves – Pathology** | | | | |
| Trigeminal neuralgia | 50% | 23% | 20% | 7% |
| Lesions of the visual pathway | 50% | 27% | 16% | 7% |
| Lesions of the oculomotor, trochlear and abducens nerve | 43% | 32% | 18% | 7% |
| Facial nerve lesions and Bell palsy | 64% | 25% | 11% | 0% |
| Lesions of glossopharyngeal nerve | 28% | 42% | 21% | 9% |
| Vertigo | 55% | 25% | 16% | 4% |
| Nystagmus | 48% | 23% | 22% | 7% |
| **Cranial Nerves – Examination** | | | | |
| Smell test – CN I | 36% | 20% | 23% | 21% |
| Confrontation test – CN II | 39% | 23% | 22% | 16% |
| Light reflexes – CN III | 45% | 23% | 18% | 14% |
| Accommodation reflexes – CN III | 43% | 25% | 18% | 14% |
| Corneal reflex – CN V | 36% | 23% | 20% | 21% |
| Jaw jerk reflex – CN V | 34% | 23% | 27% | 16% |
| Facial sensation – CN V | 50% | 27% | 14% | 9% |
| Blink reflex – CN VII | 36% | 32% | 14% | 18% |
| Stapedial reflex – CN VII | 18% | 25% | 32% | 25% |
| Lacrimal reflex – CN VII | 18% | 23% | 30% | 29% |
| Pupillary skin reflex – CN II, III, V and VII | 20% | 20% | 30% | 30% |
| Hearing test – CN VIII | 36% | 25% | 21% | 18% |
| Balance test – CN VIII | 53% | 25% | 14% | 9% |
| Carotid sinus – CN IX | 18% | 32% | 23% | 27% |
| Salivation and swallowing reflexes – CN IX | 23% | 31% | 23% | 23% |
| Elevation of soft palate – CN IX and X | 32% | 27% | 20% | 21% |
| Cough – CN X | 39% | 29% | 14% | 18% |
| Test sternocleidomastoid – CN XI | 52% | 23% | 16% | 9% |
| Tongue protrusion – CN XII | 43% | 27% | 18% | 12% |
| **Special Senses – Vision** | | | | |
| Orbit | 43% | 43% | 10% | 5% |
| Eye/eyeball | 45% | 43% | 5% | 7% |
| Sclera | 27% | 25% | 32% | 16% |
| Conjunctiva | 23% | 27% | 32% | 18% |
| Cornea | 34% | 36% | 16% | 14% |
| Uvea | 20% | 25% | 30% | 25% |
| Choroid | 21% | 18% | 34% | 27% |
| Ciliary body | 23% | 23% | 34% | 20% |
| Iris | 25% | 20% | 34% | 21% |
| Retina | 43% | 32% | 16% | 9% |
| Lens | 34% | 39% | 18% | 9% |
| Optic disc | 30% | 34% | 20% | 16% |
| Macula | 32% | 39% | 16% | 13% |
| Aqueous humor | 16% | 39% | 25% | 20% |
| Vitreous body | 16% | 34% | 27% | 23% |
| Fovea centralis | 32% | 34% | 16% | 18% |
| Suspensory ligament of eyeball | 14% | 27% | 36% | 23% |
| **Special Senses – Vision – Pathology** | | | | |
| Myopia (short-sightedness) | 23% | 23% | 31% | 23% |
| Hypermetropia (long-sightedness) | 23% | 23% | 31% | 23% |
| Astigmatism | 11% | 27% | 23% | 39% |
| Ocular trauma | 16% | 20% | 32% | 32% |
| Cataract | 14% | 25% | 36% | 25% |
| Glaucoma | 16% | 20% | 39% | 25% |
| Diabetic retinopathy | 30% | 18% | 29% | 23% |
| **Special Senses – Hearing and Balance** | | | | |
| Ear | 59% | 27% | 12% | 2% |
| External ear | 50% | 27% | 18% | 5% |
| Auricle | 39% | 32% | 25% | 4% |
| Middle ear | 59% | 30% | 7% | 4% |
| Tympanic membrane | 64% | 20% | 14% | 2% |
| Malleus | 41% | 30% | 25% | 4% |
| Incus | 41% | 30% | 25% | 4% |
| Stapes | 41% | 30% | 25% | 4% |
| Auditory tube | 45% | 20% | 30% | 5% |
| Vestibular window | 36% | 27% | 21% | 16% |
| Internal ear | 59% | 20% | 14% | 7% |
| Bony labyrinth | 48% | 25% | 18% | 9% |
| Vestibule | 55% | 20% | 16% | 9% |
| Utricle | 48% | 25% | 18% | 9% |
| Saccule | 48% | 25% | 18% | 9% |
| Maculae | 41% | 25% | 23% | 11% |
| Semicircular canals | 61% | 21% | 11% | 7% |
| Semicircular ducts | 52% | 18% | 23% | 7% |
| Ampulla of semicircular ducts | 41% | 27% | 23% | 9% |
| Perilymph | 41% | 27% | 18% | 14% |
| Cochlear | 37% | 27% | 27% | 9% |
| Scala vestibuli | 27% | 25% | 27% | 21% |
| Scala tympani | 27% | 25% | 27% | 21% |
| Cochlear duct | 34% | 27% | 25% | 14% |
| Basilar membrane | 25% | 34% | 25% | 16% |
| Spiral organ | 25% | 27% | 32% | 16% |
| Mastoid air cells | 18% | 27% | 39% | 16% |
| Cochlear ganglion | 30% | 27% | 27% | 16% |
| Cochlear nerve | 45% | 30% | 20% | 5% |
| Anterior cochlear nuclei | 23% | 25% | 34% | 18% |
| Posterior cochlear nuclei | 23% | 23% | 36% | 18% |
| Superior olivary nucleus | 20% | 20% | 46% | 14% |
| Vestibular apparatus | 52% | 27% | 14% | 7% |
| Vestibular ganglia | 32% | 23% | 32% | 13% |
| Vestibular nuclei | 43% | 25% | 23% | 9% |
| Vestibulo-ocular reflex | 59% | 14% | 16% | 11% |
| **Special Senses – Hearing and Balance – Pathology** | | | | |
| Motion sickness | 27% | 23% | 32% | 18% |
| Acute otitis media | 21% | 18% | 50% | 11% |
| External otitis | 18% | 14% | 50% | 18% |
| Mastoiditis | 20% | 16% | 41% | 23% |
| Vertigo | 50% | 23% | 20% | 7% |

**Table S6. Motor Control and Sensation, and the Autonomic Nervous System**

| **Topic** | **Essential** | **Important** | **Acceptable** | **Not Required** |
| --- | --- | --- | --- | --- |
| **Motor Control** | | | | |
| ***Motor neurons*** | 98% | 0% | 2% | 0% |
| Upper motor neuron (cell body located in the brainstem or cortex; does not have an axon in the peripheral nervous system) | 93% | 5% | 0% | 2% |
| Lower motor neuron (cell body located in the brainstem or spinal cord; has an axon in the peripheral nervous system innervating muscle) | 93% | 5% | 0% | 2% |
| ***Pyramidal motor pathways*** | 93% | 2% | 5% | 0% |
| ***Corticospinal tract*** | 93% | 5% | 2% | 0% |
| Anterior corticospinal tract | 84% | 9% | 5% | 2% |
| Lateral corticospinal tract | 91% | 7% | 0% | 2% |
| Function: Motor control of skeletal muscles in the body | 93% | 5% | 2% | 0% |
| Pathway: Cerebral cortex to spinal cord | 91% | 5% | 2% | 2% |
| ***Corticonuclear fibers (corticobulbar tract)*** | 82% | 7% | 9% | 2% |
| Function: Motor control of skeletal muscles of face, head and neck | 82% | 9% | 7% | 2% |
| Pathway: Cerebral cortex to brainstem | 80% | 9% | 7% | 4% |
| ***Extrapyramidal motor pathways*** | 75% | 16% | 9% | 0% |
| ***Tectospinal tract*** | 61% | 18% | 14% | 7% |
| Function: Reflexive postural movements in response to visual stimuli | 64% | 23% | 9% | 4% |
| Pathway: From superior colliculus of midbrain to spinal cord | 55% | 27% | 9% | 9% |
| ***Vestibulospinal tracts*** | 70% | 25% | 5% | 0% |
| Function: Facilitate activity of extensor muscles and inhibits activity of flexor muscles to contribute to the maintenance of balance | 68% | 23% | 7% | 2% |
| Pathway: From vestibular nuclei in medulla and pons to spinal cord | 61% | 25% | 9% | 5% |
| ***Rubrospinal tract*** | 59% | 20% | 16% | 5% |
| Function: Facilitates activity of flexor muscles and inhibits activity of extensor or antigravity muscles | 64% | 16% | 16% | 4% |
| Pathway: From red nucleus to spinal cord | 52% | 23% | 14% | 11% |
| ***Reticulospinal tract*** | 64% | 20% | 14% | 2% |
| Function: May facilitate or inhibit voluntary movement and reflex activity, as well as control sympathetic and parasympathetic outflow | 64% | 20% | 14% | 2% |
| Pathway: From pontine reticular formation to spinal cord | 55% | 20% | 18% | 7% |
| Pathways from cerebellum through superior peduncle to contralateral red nucleus to cortex | 23% | 32% | 35% | 10% |
| Pathways from inferior olive to contralateral cerebellum via inferior peduncle | 23% | 25% | 42% | 10% |
| Descending fibers from all areas of cortex through the crus cerebri to pons; with decussation into cerebellum via the middle peduncle | 35% | 30% | 25% | 10% |
| **Motor Control – Concepts** | | | | |
| Voluntary movement occurs in three steps: Planning, programming and execution | 93% | 5% | 2% | 0% |
| Motor homunculus in primary motor cortex | 91% | 5% | 4% | 0% |
| Spinal reflex arc | 93% | 5% | 2% | 0% |
| Neuromuscular junction | 91% | 0% | 9% | 0% |
| Motor unit | 89% | 7% | 4% | 0% |
| Open and closed loop control | 75% | 14% | 11% | 0% |
| Clinical examination of motor control (e.g., testing spinal reflexes, muscle tone and spasticity, coordination etc.) | 84% | 5% | 11% | 0% |
| **Motor Control – Pathology** | | | | |
| Upper motor neuron lesions and their consequences | 89% | 7% | 4% | 0% |
| Lower motor neuron lesions and their consequences | 89% | 7% | 4% | 0% |
| Paresis | 77% | 14% | 7% | 2% |
| Hemiparesis | 77% | 12% | 9% | 2% |
| Hemiplegia | 77% | 14% | 7% | 2% |
| Paralysis | 77% | 12% | 9% | 2% |
| Spasticity | 77% | 12% | 9% | 2% |
| Rigidity | 77% | 7% | 14% | 2% |
| Flaccidity | 77% | 7% | 16% | 0% |
| Hypotonia | 77% | 9% | 14% | 0% |
| Hypertonia | 77% | 9% | 14% | 0% |
| Dystonia | 75% | 11% | 14% | 0% |
| Tremors | 73% | 11% | 16% | 0% |
| Muscle atrophy | 80% | 9% | 11% | 0% |
| **Sensation** | | | | |
| ***Sensory neurons*** | 98% | 0% | 2% | 0% |
| First order (primary) afferents (i.e., sensory neurons with cell body located in dorsal root ganglion) | 91% | 5% | 2% | 2% |
| Second order (secondary) afferents (i.e., sensory neurons with cell body located in spinal cord/brainstem with axons projecting into the thalamus) | 87% | 9% | 2% | 2% |
| Third order afferents (i.e., sensory neurons with cell bodies located in thalamus with axons projecting to sensory cortex) | 87% | 9% | 2% | 2% |
| ***Medial lemniscus/dorsal column pathway*** | 96% | 2% | 2% | 0% |
| Function: Carries discriminative sensation (discriminatory touch and vibration), and conscious proprioception | 93% | 5% | 0% | 2% |
| Pathway: Gracile fasciculus carries input from lower half of body to gracile nucleus and cuneate fasciculus carries input from upper half of body to cuneate nucleus | 82% | 11% | 5% | 2% |
| ***Lateral spinothalamic tract*** | 93% | 5% | 2% | 0% |
| Function: Carries nociception and temperature | 89% | 9% | 0% | 2% |
| Pathway: To thalamus along lateral spinothalamic tracts | 77% | 21% | 0% | 2% |
| ***Anterior spinothalamic tract*** | 84% | 9% | 7% | 0% |
| Function: Carries crude touch and pressure | 80% | 14% | 4% | 2% |
| Pathway: To thalamus along anterior spinothalamic tract | 68% | 23% | 5% | 4% |
| ***Spinocerebellar tracts*** | 77% | 12% | 9% | 2% |
| Function: Carry unconscious proprioceptive information | 75% | 11% | 12% | 2% |
| Pathway: Along posterior spinocerebellar tract which passes through the posterior thoracic nucleus and accessory cuneate nucleus or anterior spinocerebellar tract to cerebellum | 55% | 27% | 14% | 4% |
| ***Cuneocerebellar tract*** | 28% | 20% | 45% | 7% |
| Function: Carries unconscious proprioceptive information from the upper limbs | 30% | 20% | 45% | 5% |
| ***Posterior spinocerebellar tract*** | 33% | 25% | 37% | 5% |
| Function: Carries unconscious proprioceptive information from the trunk and lower limbs | 35% | 23% | 37% | 5% |
| ***Sensory receptors*** | 75% | 14% | 11% | 0% |
| Mechanoreceptor – Meissner corpuscles | 52% | 27% | 16% | 5% |
| Function of Meissner corpuscles: Sense fine touch or discriminative sensation | 57% | 23% | 16% | 4% |
| Mechanoreceptor – Pacinian corpuscles | 57% | 23% | 13% | 7% |
| Function of Pacinian corpuscles: Sense pressure and vibration | 59% | 20% | 16% | 5% |
| Free nerve terminals | 61% | 20% | 14% | 5% |
| Function of free nerve terminals: thermoreception (temperature) and nociception | 66% | 16% | 14% | 4% |
| Proprioceptive and position sense receptor – muscle spindles | 68% | 16% | 11% | 5% |
| Intrafusal muscle fibers | 64% | 18% | 14% | 4% |
| Proprioceptive and position sense receptor – Golgi tendon organs | 70% | 16% | 9% | 5% |
| Proprioceptive and position sense receptor – joint receptors | 70% | 16% | 9% | 5% |
| **Sensation – Concepts** | | | | |
| Sensory modalities (e.g., discriminatory and crude touch, conscious and unconscious proprioception, nociception, vibration sense, etc.) | 89% | 7% | 2% | 2% |
| Conscious (e.g., discriminatory touch) and unconscious (e.g., unconscious proprioceptive) sensory information | 84% | 9% | 7% | 0% |
| Pain | 89% | 4% | 7% | 0% |
| Somatic pain | 82% | 11% | 7% | 0% |
| Visceral pain | 75% | 18% | 7% | 0% |
| Pain vs nociception | 80% | 9% | 9% | 2% |
| Peripheral and central sensitization | 73% | 14% | 9% | 4% |
| Pain as a protective output of the CNS | 73% | 16% | 9% | 2% |
| Relationship between neural plasticity and persistent pain states | 73% | 9% | 14% | 4% |
| Sensory homunculus in primary somatosensory cortex | 82% | 7% | 9% | 2% |
| Clinical examination of sensation | 75% | 9% | 11% | 5% |
| **Sensation – Pathology** | | | | |
| Understand lesions of sensory pathways and their consequences | 89% | 5% | 4% | 2% |
| Persistent pain | 70% | 14% | 9% | 7% |
| Phantom limb | 64% | 14% | 20% | 2% |
| Referred pain | 77% | 14% | 7% | 2% |
| **Somatic Plexi** | | | | |
| Cervical plexus – please note, all other plexuses have been included in the musculoskeletal syllabus | 57% | 13% | 23% | 7% |
| **Autonomic Nervous System** | | | | |
| ***Nerve plexi*** | 39% | 25% | 29% | 7% |
| Cardiac plexus | 9% | 39% | 41% | 11% |
| Pulmonary plexus | 9% | 34% | 43% | 14% |
| Esophageal plexus | 9% | 27% | 39% | 25% |
| Abdominal aortic plexus | 9% | 27% | 39% | 25% |
| Celiac plexus | 9% | 29% | 39% | 23% |
| Superior hypogastric plexus | 5% | 27% | 41% | 27% |
| Inferior hypogastric plexus | 5% | 27% | 41% | 27% |
| Superior mesenteric plexus | 9% | 29% | 39% | 23% |
| ***Parasympathetic nervous system*** | 70% | 16% | 14% | 0% |
| Preganglionic cell bodies associated with CN III, VII, IX and X and lateral horns of S2–S4 segments of spinal cord | 55% | 25% | 18% | 2% |
| Postganglionic cell bodies within or close to target organ | 41% | 27% | 25% | 7% |
| Ciliary ganglion | 23% | 20% | 43% | 14% |
| Pterygopalatine ganglion | 11% | 23% | 43% | 23% |
| Otic ganglion | 14% | 20% | 43% | 23% |
| Pelvic splanchnic nerves | 20% | 27% | 37% | 16% |
| ***Sympathetic nervous system (SNS)*** | 75% | 14% | 11% | 0% |
| Preganglionic cell bodies in lateral horns of T1-L2 segments of spinal cord | 55% | 25% | 18% | 2% |
| Sympathetic trunk | 50% | 25% | 25% | 0% |
| Paravertebral ganglia | 39% | 25% | 27% | 9% |
| Prevertebral ganglia | 36% | 27% | 28% | 9% |
| Pathways for SNS to exit the sympathetic trunk | 32% | 29% | 32% | 7% |
| Superior cervical ganglion | 23% | 23% | 43% | 11% |
| Middle cervical ganglion | 20% | 18% | 46% | 16% |
| Inferior cervical ganglion | 20% | 25% | 39% | 16% |
| Cardiopulmonary splanchnic nerves | 16% | 18% | 43% | 23% |
| Abdominopelvic splanchnic nerves | 14% | 18% | 43% | 25% |
| Inferior mesenteric ganglion | 9% | 23% | 41% | 27% |
| **Autonomic Nervous System – Concepts** | | | | |
| General organization of the autonomic nervous system i.e., sympathetic and parasympathetic divisions | 80% | 16% | 4% | 0% |
| Autonomic innervation of the body | 77% | 16% | 7% | 0% |
| Autonomic nervous system functions to maintain homeostasis | 77% | 16% | 7% | 0% |
| **Autonomic Nervous System – Pathology** | | | | |
| Autonomic control following spinal cord injury | 62% | 18% | 18% | 2% |
| Intermittent claudication | 39% | 25% | 27% | 9% |

*Note.* CN = cranial nerve.
